# Succinate metabolism and membrane reorganization drives the endotheliopathy and coagulopathy of traumatic hemorrhage

**DOI:** 10.1126/sciadv.adf6600

**Published:** 2023-06-14

**Authors:** Sarah Abdullah, Michael Ghio, Aaron Cotton-Betteridge, Aditya Vinjamuri, Robert Drury, Jacob Packer, Oguz Aras, Jessica Friedman, Mardeen Karim, David Engelhardt, Emma Kosowski, Kelby Duong, Farhana Shaheen, Patrick R. McGrew, Charles T. Harris, Robert Reily, Mimi Sammarco, Partha K. Chandra, Derek Pociask, Jay Kolls, Prasad V. Katakam, Alison Smith, Sharven Taghavi, Juan Duchesne, Olan Jackson-Weaver

**Affiliations:** ^1^ Department of Surgery, Tulane University School of Medicine, New Orleans, LA, USA.; ^2^ Tulane University School of Medicine, New Orleans, LA, USA.; ^3^ Loyola University, New Orleans, LA, USA.; ^4^ Tulane University, New Orleans, LA, USA.; ^5^ Tulane University School of Medicine, Center for Translational Research in Infection and Inflammation, New Orleans, LA, USA.; ^6^ Louisiana State University Health Sciences Center, New Orleans, LA, USA.; ^7^ University Medical Center, New Orleans, LA, USA.; ^8^ Department of Pharmacology, Tulane University School of Medicine, New Orleans, LA, USA.

## Abstract

Acute hemorrhage commonly leads to coagulopathy and organ dysfunction or failure. Recent evidence suggests that damage to the endothelial glycocalyx contributes to these adverse outcomes. The physiological events mediating acute glycocalyx shedding are undefined, however. Here, we show that succinate accumulation within endothelial cells drives glycocalyx degradation through a membrane reorganization-mediated mechanism. We investigated this mechanism in a cultured endothelial cell hypoxia-reoxygenation model, in a rat model of hemorrhage, and in trauma patient plasma samples. We found that succinate metabolism by succinate dehydrogenase mediates glycocalyx damage through lipid oxidation and phospholipase A2-mediated membrane reorganization, promoting the interaction of matrix metalloproteinase 24 (MMP24) and MMP25 with glycocalyx constituents. In a rat hemorrhage model, inhibiting succinate metabolism or membrane reorganization prevented glycocalyx damage and coagulopathy. In patients with trauma, succinate levels were associated with glycocalyx damage and the development of coagulopathy, and the interaction of MMP24 and syndecan-1 was elevated compared to healthy controls.

## Teaser

Endothelial metabolism is altered in traumatic hemorrhage leading to membrane reorganization and glycocalyx shedding.

## INTRODUCTION

The endothelial glycocalyx (EG) is a complex protein and carbohydrate structure that lines the luminal surface of the endothelium. Although electron microscopy has allowed visualization of this structure under certain conditions for decades (*1*), a full appreciation of its regulation and involvement in disease processes has been slow in coming. Functionally, the EG prevents inappropriate activation of coagulation cascades at the interface between blood and endothelial cell surface (*2*–*4*). The glycocalyx also regulates vascular permeability (*5*–*7*), endothelial response to shear stress (*8*), leukocyte interactions with the endothelial surface (*7*), and may act as a dynamic reservoir for a variety of signaling molecules (*9*). Recently, increasing interest in the glycocalyx damage that occurs during a broad range of critical illnesses has developed, due to a proliferation of studies that report increases in shed glycocalyx components in the plasma and the association of this phenomenon with poor outcomes (*3*, *4*, *7*, *10*–*16*). This damage to the EG in concert with general endothelial dysfunction has been termed “endotheliopathy.” Glycocalyx shedding is strongly associated with the coagulopathy that occurs in critical illness (*13*). This is thought to be due to both autoheparinization of the blood, as well as a consumptive coagulopathy due to activation of the coagulation cascade on the unprotected endothelial surface (*3*, *4*, *11*, *12*). Coagulopathy is a common complication that occurs after substantial hemorrhage, confounding attempts to achieve hemostasis and markedly increasing mortality. Counteracting coagulopathy by administering blood products or protein constituents is helpful (*17*, *18*), but persistent coagulopathy after massive hemorrhage contributes to many deaths per year, highlighting the need for treatments based on a deeper knowledge of fundamental mechanisms.

Although EG shedding is thought to be enzymatically mediated (*11*, *19*), a mechanistic understanding of this process has not been elucidated. In particular, the events leading to acute glycocalyx damage, which occurs too rapidly to be accounted for by transcriptional alterations, are unknown.

We have previously demonstrated that mitochondrial reactive oxygen species (ROS) mediate EG damage in vitro during hypoxia-reoxygenation (H-R) (*20*, *21*). Ischemia-reperfusion injury across various cell types has long been known to lead to an increase in mitochondrial ROS (mitoROS) production. The precise mechanism mediating this effect has remained elusive. Recently, it has been proposed that a buildup of the citric acid cycle metabolite succinate is a primary driver of ROS production under these conditions (*22*, *23*). Succinate has been shown to be an independent predictor of mortality in severely injured patients (*24*), suggesting that it may contribute to pathological processes in trauma. Furthermore, our group observed a beneficial effect of succinate dehydrogenase (SDH) inhibition on heart function and lung glycocalyx in a large animal hemorrhage model (*25*). However, the mechanism by which succinate affects the glycocalyx is unclear, as is whether succinate affects coagulopathy and the resulting organ damage.

Here, we hypothesize that succinate buildup and metabolism drive EG damage and the resulting coagulopathy in traumatic hemorrhage. Using evidence from cell culture, animal models, and trauma patient plasma, we propose that succinate is an important driver of EG damage. In addition, we propose that the likely mechanism is an unexpected alteration in endothelial plasma membrane organization.

## RESULTS

### Cell culture model

**Fig. 1.**
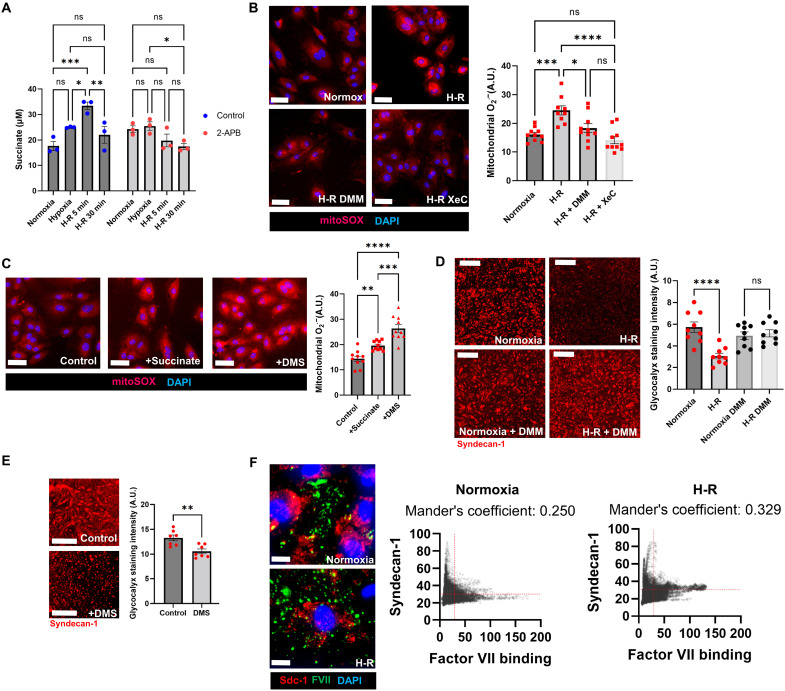
Succinate metabolism mediates mitochondrial reactive oxygen species (ROS) production and glycocalyx damage in cultured endothelial cells. (**A**) Succinate levels in human umbilical vein endothelial cells (HUVECs) during hypoxia-reoxygenation (H-R) protocol ± 2-APB (2-aminoethoxydiphenyl borate) (100 μM). *n* = 3 biological replicates. Significance was assessed with a two-way analysis of variance (ANOVA) corrected for multiple comparisons using Tukey’s method. (**B**) Mitochondrial ROS assessed by mitoSOX fluorescence in HUVECs during H-R ± xestospongin C (XeC) or dimethylmalonate (DMM). *n* = 10 biological replicates. Significance was assessed with one-way ANOVA corrected for multiple comparisons using Tukey’s method. Scale bars, 20 μm. (**C**) Mitochondrial ROS assessed by mitoSOX fluorescence in HUVECs during succinate (2 mM) or dimethylsuccinate (DMS; 50 μM) administration. *n* = 10 biological replicates. Scale bars, 20 μm. Significance was assessed with one-way ANOVA corrected for multiple comparisons using Tukey’s method. (**D**) Surface syndecan-1 immunostaining during the H-R protocol to assess glycocalyx levels in HUVECs ± DMM (50 μM). Significance was assessed with one-way ANOVA corrected for multiple comparisons using Tukey’s method. *n* = 9 biological replicates. Scale bars, 100 μm. (**E**) Surface syndecan-1 immunostaining during the H-R protocol to assess glycocalyx levels in HUVECs ± DMS (50 μM) administration. Significance was assessed with a two-tailed Student’s *t* test. *n* = 7 biological replicates. Scale bars, 100 μm. (**F**) Recombinant His-tagged human Factor VII (FVII) binding to HUVECs is excluded from syndecan-1 domains. Cytofluorograms show FVII and syndecan-1 staining intensity per pixel, demonstrating coexclusion. Dotted red lines were added to show minimal coexpression (upper right quadrant). Mander’s coefficient quantifies the overlap between the channels. Representative images and cytofluorograms from three biological replicates were shown. Error bars in all figures represent the means ± SEM. DAPI, 4′,6-diamidino-2-phenylindole; A.U. arbitrary unit; ns, not significant.

We first investigated the role of succinate in an in vitro H-R model of glycocalyx damage using human umbilical vein endothelial cells (HUVECs). We found that succinate was elevated during hypoxia and shortly after reoxygenation but was reduced to baseline levels after longer-term reoxygenation (Fig. 1A). We previously demonstrated that Ca^2+^ signaling is a necessary step in glycocalyx damage (*20*), and so we inhibited Ca^2+^ signaling using 2-aminoethoxydiphenyl borate. This prevented the buildup of succinate (Fig. 1A), suggesting that alterations in mitochondrial metabolite flux during H-R are regulated by Ca^2+^ signaling. To determine whether inositol 1,4,5-trisphosphate receptor (IP_3_R) Ca^2+^ release induces succinate buildup, we incubated HUVECs with membrane-permeant IP_3_ ester i-IP_3_/PM (*26*). I-IP3/PM elevated succinate 5 min after application, suggesting that IP_3_R activation is sufficient to mediate succinate elevations (fig. S1A). Ca^2+^ elevation occurs during hypoxia, aligning with the time course of succinate elevation (fig. S1B). Succinate metabolism to generate ROS is mediated by SDH (*23*), and this process can be prevented with the SDH inhibitor dimethylmalonate (DMM). We next assessed mitoROS using MitoSOX and found that the elevation in mitoROS during H-R was prevented by blocking IP_3_R Ca^2+^ signaling with xestospongin C or SDH with DMM (Fig. 1B). To assess whether succinate elevation is sufficient to induce mitoROS, we treated HUVECs with succinate or the more cell-permeable analog dimethylsuccinate (DMS) and found that both elevated mitoROS (Fig. 1C). These treatments did not elevate intracellular succinate beyond physiological levels (fig. S1D).

We next measured glycocalyx damage using syndecan-1 surface immunostaining (a major constituent of the glycocalyx) and found that DMM prevented glycocalyx loss during H-R (Fig. 1D). The loss of surface glycocalyx staining was associated with an increase in soluble syndecan-1 in the media, suggesting that this observation represents a true shedding event and not an endocytic or another internalization event (fig. S1E). To determine whether succinate elevation alone was sufficient to cause glycocalyx shedding, we treated HUVECs with DMS and observed a substantial loss in syndecan-1 levels (Fig. 1E).

We next investigated whether glycocalyx shedding is mediated by succinate metabolism in endothelial cells from other vascular beds. For this, we performed H-R in human dermal microvascular endothelial cells (HDMECs) and human pulmonary artery endothelial cells (HPAECs). We found that H-R induced syndecan-1 shedding in HDMECs and this was prevented with DMM (fig. S1F). H-R alone was insufficient to induce glycocalyx shedding in HPAECs (fig. S1G). We found that norepinephrine (10 μM) along with H-R did induce shedding, and this was prevented with DMM (fig. S1G). This suggests that certain vascular beds may require combined H-R insult and catecholamine stimulation to induce glycocalyx damage, but that succinate metabolism is the mediator in these cases as well.

The syndecan-1 staining in HUVECs is most prominent in the center of the cell mass but is not a nuclear stain (fig. S2A). Furthermore, the image acquisition settings were chosen so as to not overexpose areas of high syndecan-1 levels. Brighter images demonstrate that cells were 90 to 100% confluent for all syndecan-1–shedding experiments (fig. S2B).

The glycocalyx acts as a sterically based boundary layer preventing the interaction of inflammatory and coagulant proteins with the endothelial surface (*27*, *28*). To assess whether syndecan-1 staining on the endothelial surface represents a functional glycocalyx, we incubated HUVECs with recombinant coagulation factor VII (FVII) and costained for FVII and syndecan-1. Syndecan-1 areas excluded FVII binding, indicating that this represents a functional anticoagulant glycocalyx (Fig. 1F). This FVII staining occurred on the periphery of the HUVECs and was not a nonspecific binding to the plastic plate surface (fig. S3A). Furthermore, syndecan-1 excluded the binding of recombinant l-selectin (fig. S3B), a mediator of leukocyte adhesion to endothelial cells. The glycocalyx also binds and sequesters anticoagulant molecules such as antithrombin III (AT III). To assess whether the HUVECs in this study also display this aspect of glycocalyx function, we incubated the cells with recombinant AT III and costained for AT III and syndecan-1. Unexpectedly, we found that syndecan-1 excluded AT III binding (fig. S3C). However, AT III may primarily bind the syndecan-4 component of the glycocalyx (*29*). We repeated AT III incubation and costained with syndecan-4. We found that AT III colocalizes with syndecan-4 (fig. S4A). We also found that H-R reduced the level of AT III binding to HUVECs but DMM prevented this effect (fig. S4, A and B). This decrease in AT III binding during H-R was not associated with a substantial decrease in syndecan-4 staining (fig. S4B). This could potentially be mediated by nonproteolytic alterations in the glycosaminoglycan component of syndecan-4 (*30*), potentially through direct oxidation or other modifications. We next costained HUVECs for syndecan-1 and syndecan-4 to resolve the disparate results seen in AT III binding. We found that syndecan-4 is present at moderate levels in regions with high syndecan-1, but there is a separate population of syndecan-4 that exists in a punctate pattern outside of the syndecan-1 regions (fig. S4C).

**Fig. 2.**
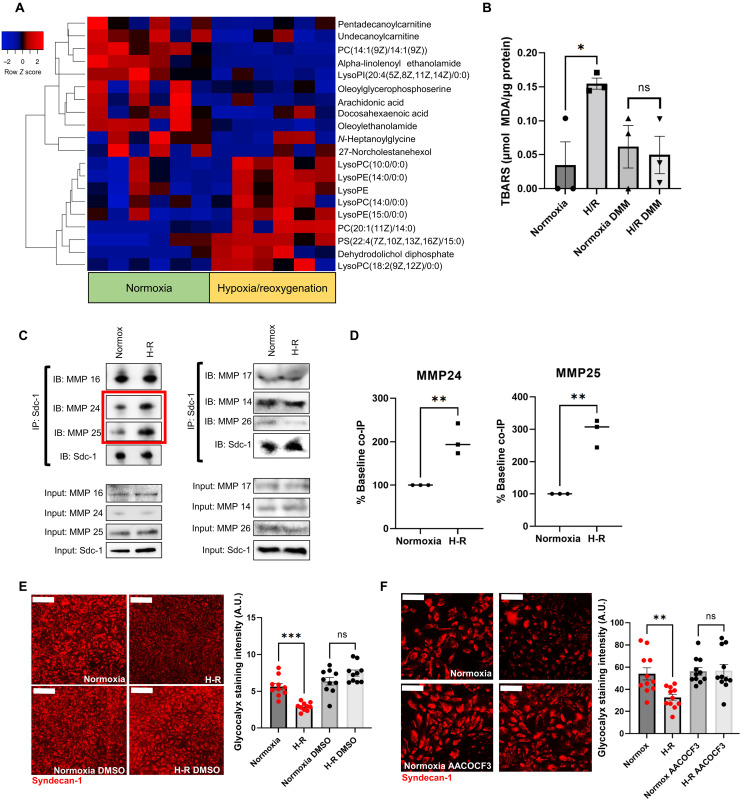
Alterations in membrane organization mediate glycocalyx damage. (**A**) Heatmap of significant lipid alterations from untargeted metabolomics analysis of human umbilical vein endothelial cells (HUVECs) during the hypoxia-reoxygenation (H-R) protocol. *n* = 6 biological replicates. *Z* scores were indicated by color, as a measure of relative abundance of the metabolite. (**B**) Lipid oxidation levels measured by thiobarbituric acid reactive substances (TBARS) assay in HUVECs subjected to the H-R protocol ± dimethylmalonate (DMM) (50 μM). *n* = 3 biological replicates. Significance was assessed with one-way analysis of variance (ANOVA) corrected for multiple comparisons using Tukey’s method. (**C**) Coimmunoprecipitation (co-IP) of syndecan-1 and integral membrane matrix metalloproteinase (MMP) binding partners in HUVECs subjected to the H-R protocol. IP = immunoprecipitation target; IB = protein probed by immunoblot; input bands = total protein before immunoprecipitation. The red box highlights proteins showing an increase in interaction after H-R. (**D**) Quantification of MMP24 and MMP25 interaction (coimmunoprecipitation, “co-IP”) with syndecan-1 in HUVECs subjected to the H-R protocol. *n* = 3 biological replicates. Significance was assessed with a two-tailed Student’s *t* test. (**E**) Surface syndecan-1 immunostaining during the H-R protocol to assess glycocalyx levels in HUVECs ± dimethyl sulfoxide (DMSO, 0.1% in media). *n* = 10 biological replicates. Significance was assessed with one-way ANOVA corrected for multiple comparisons using Tukey’s method. Scale bars, 100 μm. (**F**) Surface syndecan-1 immunostaining during the H-R protocol to assess glycocalyx levels in HUVECs ± phospholipase A2 inhibitor AACOCF3 (5 μM). *n* = 11 biological replicates. Significance was assessed with one-way ANOVA corrected for multiple comparisons using Tukey’s method. Scale bars, 50 μm. Error bars in all figures represent the means ± SEM. A.U., arbitrary units.

To determine additional metabolic alterations occurring during H-R of endothelial cells, we performed untargeted metabolomics of cell lysates. ɣ-Aminobutyric acid (GABA) was elevated by hypoxia (fig. S5), suggesting that the GABA shunt pathway (*31*) may be the source of succinate. Unexpectedly, in the metabolomics analysis, we found that membrane lipids were substantially altered by H-R. Specifically, several species of lysophospholipids were increased after H-R (Fig. 2A). Lysophospholipids are produced by phospholipase activity (*32*), and these enzymes preferentially cleave oxidized lipids (*33*, *34*). To determine whether lipid oxidation occurs during H-R, we performed a thiobarbituric acid reactive substances (TBARS) assay. We found that H-R increases lipid oxidation, but this was prevented by inhibiting succinate metabolism with DMM (Fig. 2B).

We next performed a series of protease activity assays to assess whether this alteration in membrane lipids led to an increase in extracellular protease activity. We incubated HUVECs under normoxic or H-R conditions with either *N*α-Benzoyl-dl-arginine 4-nitroanilide hydrochloride (BAPNA), quenched fluorescent casein, or quenched fluorescent gelatin. There was no increase in extracellular protease activity with these assays (fig. S4), and we observed a tendency for decreased overall protease activity. We also performed specific assays for the enzymes matrix metalloproteinase 7 (MMP7), MMP14, a disintegrin and metalloproteinase 17 (ADAM17), and MMP3, known to function as sheddases in other contexts (*19*, *35*). Similar to the broad-spectrum protease activity, these enzyme activities in the extracellular space were not increased during H-R but showed a tendency to decrease instead (fig. S6). Gelatin zymography of the media from these cells also showed a decrease in MMP2 and MMP9 activity (fig. S6H). This suggests that EG degradation is due to spatial regulation of existing surface proteases, rather than an increase in protease activity generally in the extracellular space.

Membrane lipid oxidation and increases in lysophospholipids can alter membrane organizational properties (*36*–*39*). We next performed a series of immunoprecipitation experiments to determine whether these lipid alterations affected the interaction of syndecan-1 with integral membrane MMPs. We focused on integral membrane MMPs because the distribution of these enzymes would be most affected by changes in membrane organization. We found that the interaction of syndecan-1 with MMP24 and MMP25 was increased after H-R (Fig. 2, C and D), suggesting that elevations in oxidized lipids and lysophospholipids could increase the association of sheddase enzymes with glycocalyx constituents. We next investigated whether alterations in membrane organization per se lead to glycocalyx degradation, rather than membrane lipid alterations leading to signaling events that promoted glycocalyx degradation. We incubated HUVECs with 0.1% dimethyl sulfoxide (DMSO), which reduces membrane organization (*40*, *41*), during normoxia or H-R. We observed that DMSO prevented glycocalyx degradation (Fig. 2E), suggesting that altered membrane organization domains were necessary for glycocalyx shedding.

To determine whether phospholipase A2 (PLA2) activity, which is the primary enzyme responsible for lipoprotein lipase (LPL) generation (*32*), was required for glycocalyx shedding, we treated HUVECs with arachidonyl trifluoromethyl ketone (AACOCF3), an inhibitor of 85-kDa cytosolic PLA2. We found that AACOCF3 prevented glycocalyx shedding during H-R, which suggests that the production of LPLs is necessary for shedding (Fig. 2F). We performed SDS–polyacrylamide gel electrophoresis (PAGE) on cell lysates to measure PLA2 phosphorylation. We found that PLA2 phosphorylation was not increased by H-R, suggesting that PLA2-mediated increases in LPL generation are likely due to preferential cleavage of oxidized lipids (fig. S7A). To determine whether an increase in LPLs was sufficient to cause glycocalyx shedding, we incubated HUVECs with lysophosphatidylcholine (LPC) (1 ng/μl) for 1.5 hours. This induced general glycocalyx damage, as measured by wheat germ agglutinin (WGA) staining (fig. S7B), as well as specific syndecan-1 shedding (fig. S7C). Demonstrating that this effect was due to membrane organization effects, DMSO prevented the LPL-induced glycocalyx damage (fig. S7B).

**Fig. 3.**
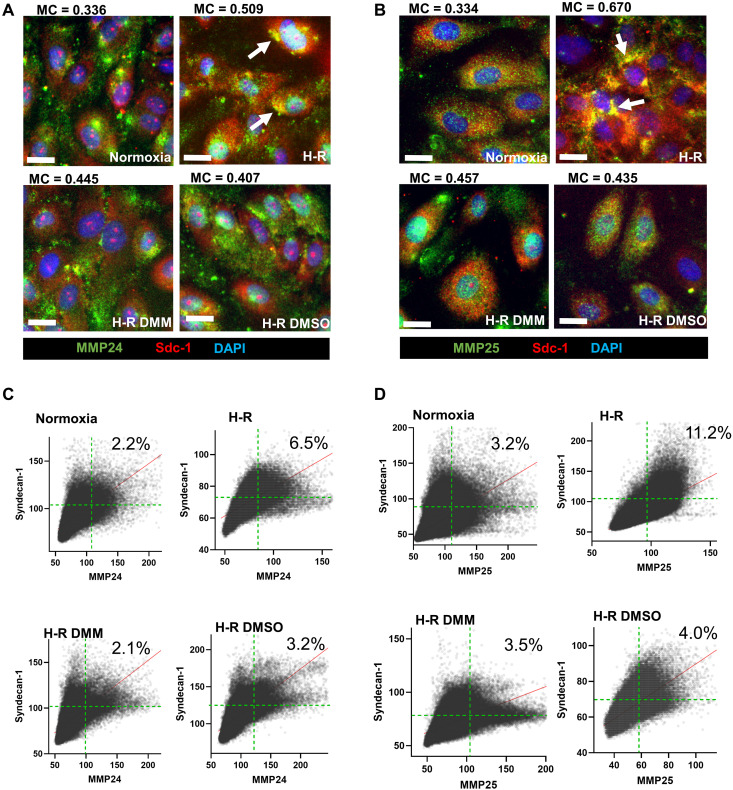
Hypoxia-reoxygenation induces colocalization of matrix metalloproteinases (MMPs) and syndecan-1 through succinate metabolism and membrane organization effects. (**A**) Immunofluorescence staining of surface MMP24 and syndecan-1 in human umbilical vein endothelial cells (HUVECs) subjected to the hypoxia-reoxygenation (H-R) protocol. Representative images from three biological replicates were shown. Arrows indicate the areas of high MMP24 and syndecan-1 staining. Mander’s coefficient quantifies the overlap between the channels. (**B**) Immunofluorescence staining of MMP25 and syndecan-1 in HUVECs subjected to the H-R protocol. Representative images from three biological replicates were shown. Arrows indicate the areas of high MMP25 and syndecan-1 staining. Mander’s coefficient quantifies the overlap between the channels. Scale bars, 20 μm. (**C**) Cytofluorograms of pixel intensity of MMP24 and syndecan-1 staining in experiments from (A). Numbers indicate the percentage of total pixels in the upper right quadrants, corresponding to high MMP24 and syndecan-1 fluorescence intensity in single pixels. Representative plots from three biological replicates were shown. (**D**) Cytofluorograms of pixel intensity of MMP25 and syndecan-1 staining in experiments from (B). Numbers indicate the percentage of total pixels in the upper right quadrants, corresponding to high MMP25 and syndecan-1 fluorescence intensity in corresponding single pixels. Representative plots from three biological replicates were shown.

We then performed immunofluorescence to visualize whether MMP24 and MMP25 distribution was altered by H-R. We found that MMP24 and MMP25 became concentrated in specific subregions of the cells after H-R, and we observed that syndecan-1 was also elevated in these regions (Fig. 3, A to D). Treatment of the cells with DMM or DMSO prevented this reorganization.

### Hemorrhagic shock

**Fig. 4.**
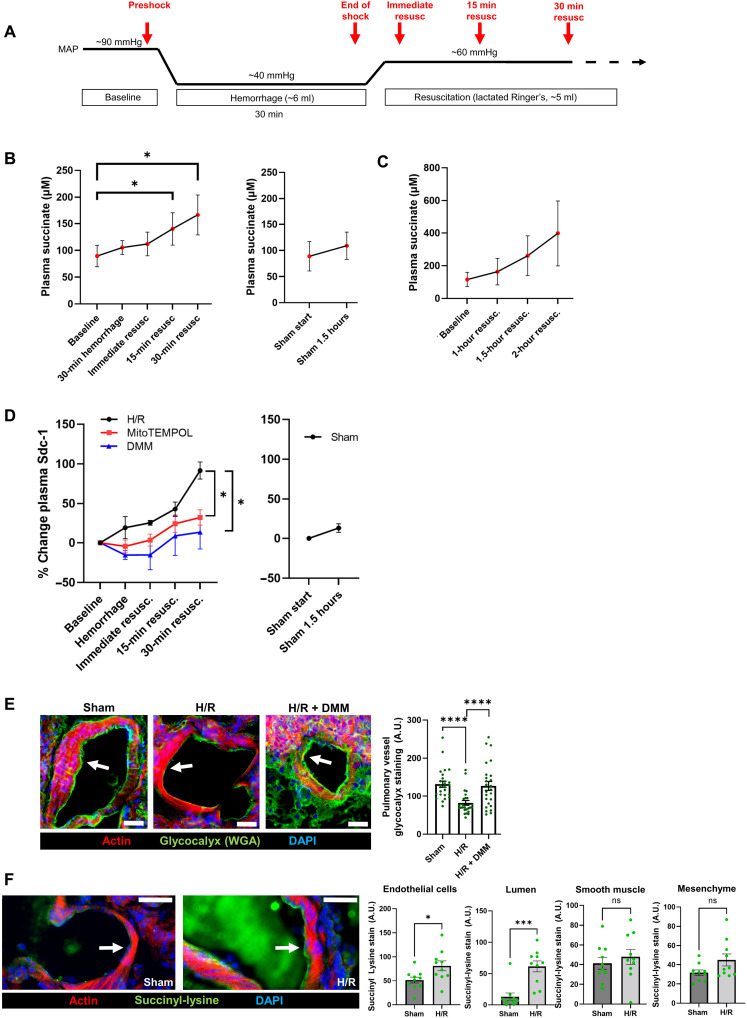
Succinate metabolism mediates glycocalyx damage in vivo. (**A**) Protocol outline for hemorrhage/resuscitation (H/R) in rats. Red arrows indicate blood sampling time points. MAP, mean arterial pressure. (**B**) Plasma succinate measurements in H/R or sham control rats. *n* = 5 biological replicates for H/R, 3 biological replicates for sham. Significance was assessed with one-way repeated measures analysis of variance (ANOVA) corrected for multiple comparisons using Tukey’s method. (**C**) Plasma succinate measurements in H/R rats at longer time points. (**D**) Plasma syndecan-1 measurements in H/R or sham control rats ± mitoTEMPOL (5 mg/kg) or dimethylmalonate (DMM) (50 mg/kg). *n* = 5 biological replicates per group. Significance was assessed with two-way repeated measures ANOVA corrected for multiple comparisons using Tukey’s method. Values were expressed as the percent change from the baseline. No differences in the baseline were present (fig. S8A). (**E**) Lung vessel glycocalyx assessed by fluorescent wheat germ agglutinin (WGA) staining in H/R or sham control rats ± DMM (50 mg/kg). Actin stain (fluorescent phalloidin) was used to aid in vessel identification. *n* = 25 vessels from four to five rats per treatment group. Significance was assessed with one-way ANOVA corrected for multiple comparisons using Tukey’s method. Arrows indicate glycocalyx. Scale bars, 20 μm. (**F**) Lung vessel succinyl-lysine level assessed by immunofluorescence staining in H/R or sham control rats. Succinyl-lysine staining intensity was quantified in endothelial cells, vessel lumen, smooth muscle cells, and mesenchyme. *n* = 10 vessels from four rats per group. Actin stain (fluorescent phalloidin) was used to aid in vessel identification. Arrows indicate endothelial cells. Scale bars, 20 μm. Significance was assessed with a two-tailed Student’s *t* test. Error bars in all figures represent the means ± SEM. A.U., arbitrary units.

We next performed experiments in a rat model of hemorrhagic shock (Fig. 4A) to test the role of succinate in mediating glycocalyx damage in vivo. We found that succinate in the plasma was increased in this model after resuscitation (Fig. 4, B and C). We collected serial plasma samples to measure syndecan-1 as a marker of glycocalyx damage. We found that syndecan-1 was significantly elevated during resuscitation, but that mitochondrial superoxide scavenger MitoTEMPOL (2,2,6,6-Tetramethyl-4-{[5-(triphenylphosphonio)pentyl]oxy}-1-piperidinyloxy bromide) or DMM prevented this (Fig. 4D). This syndecan-1 shedding was independent of resuscitation fluid used (fig. S8B). MitoTEMPOL or DMM did not affect the volume of bloodshed to achieve the target mean arterial pressure (MAP) or the volume of fluid needed during resuscitation.

We measured glycocalyx in lung vasculature by lectin staining, using fluorescent WGA. Syndecan-1 staining is ideal for cultured cells, where fine structure can be resolved, but in vivo a more general measurement of the glycocalyx is preferable. Therefore, we chose to quantify total glycocalyx with WGA. Furthermore, we found that WGA staining intensity is more sensitive to changes than measurements of the thickness of the fluorescent layer. Average traces of sham and H/R pulmonary vessels show a large decrease in fluorescence intensity, whereas the width of the layer at half maximum intensity is only modestly affected (fig. S8C). Using these methods, we found that hemorrhage/resuscitation (H/R) caused loss of lung vessel glycocalyx, but that treatment with DMM during the initiation of resuscitation prevented this shedding (Fig. 4E). These glycocalyx measurements were confirmed in living pulmonary artery segments isolated immediately after the resuscitation period (fig. S8D).

To assess whether succinate levels in endothelial cells were elevated during H/R, we immunostained lung sections for succinyl-lysine, a posttranslational modification of proteins that is elevated by increases in intracellular succinate concentration (fig. S9A) (*42*). We observed that succinyl-lysine staining in endothelial cells and in the vessel lumen was increased in H/R, indicating that succinate in endothelial cells and plasma was likely elevated (Fig. 4F). Succinyl-lysine levels also could be affected by desuccinylase enzymes, the primary one being sirtuin 5 (SIRT5) (*43*). We quantified endothelial SIRT5 using immunofluorescence in pulmonary vessels and did not observe a change in H/R-treated rats (fig. S9B). This suggests that succinyl-lysine staining levels likely do reflect succinate levels.

**Fig. 5.**
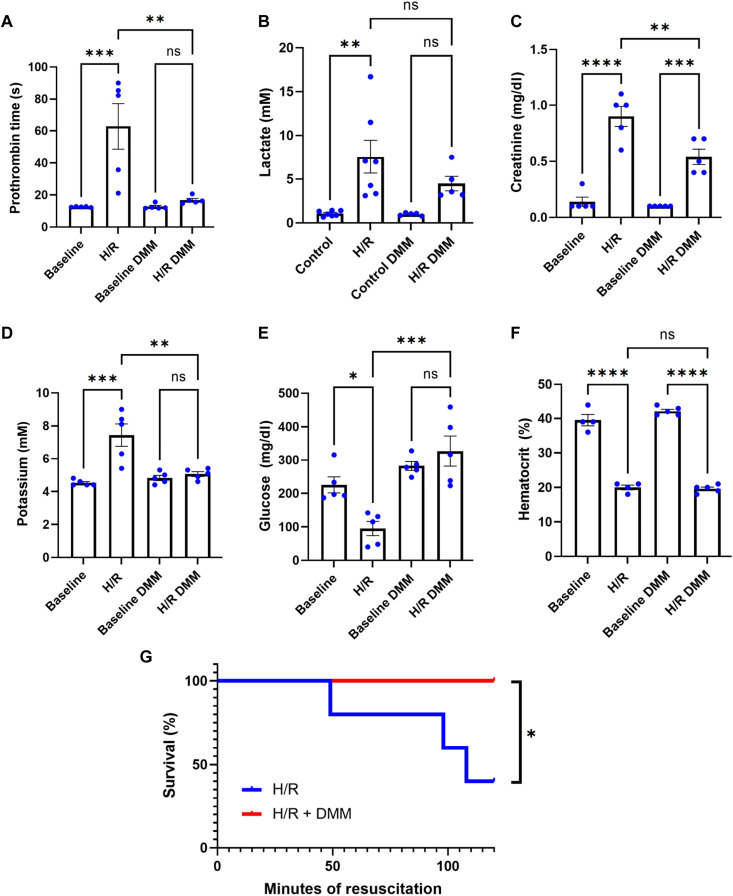
Inhibition of succinate dehydrogenase prevents coagulopathy and organ dysfunction after hemorrhage. (**A**) Assessment of coagulation using prothrombin time (PT) in blood from hemorrhage/resuscitation (H/R) rats ± dimethylmalonate (DMM) (50 mg/kg) treatment at baseline and after 90 min of resuscitation. Significance was assessed with one-way analysis of variance (ANOVA) corrected for multiple comparisons using Tukey’s method. *n* = 5 animals per group. (**B**) Lactate levels in blood from H/R rats ± DMM (50 mg/kg) treatment at baseline and after 90 min of resuscitation. Significance was assessed with one-way ANOVA corrected for multiple comparisons using Tukey’s method. *n* = 5 to 7 animals per group. (**C**) Creatinine levels in blood from H/R rats ± DMM (50 mg/kg) treatment at baseline and after 90 min of resuscitation. Significance was assessed with one-way ANOVA corrected for multiple comparisons using Tukey’s method. *n* = 5 animals per group. (**D**) Potassium levels in blood from H/R rats ± DMM (50 mg/kg) treatment at baseline and after 90 min of resuscitation. Significance was assessed with one-way ANOVA corrected for multiple comparisons using Tukey’s method. *n* = 5 animals per group. (**E**) Glucose levels in blood from H/R rats ± DMM (50 mg/kg) treatment at baseline and after 90 min of resuscitation. Significance was assessed with one-way ANOVA corrected for multiple comparisons using Tukey’s method. *n* = 5 animals per group. (**F**) Hematocrit levels from H/R rats ± DMM (50 mg/kg) treatment at baseline and after 90 min of resuscitation. Significance was assessed with one-way ANOVA corrected for multiple comparisons using Tukey’s method. *n* = 4 to 5 animals per group. (**G**) Kaplan-Meier survival curves for H/R rats ± DMM (50 mg/kg). Log-rank (Mantel-Cox) test was used to assess significance. *N* = 5 animals per group. Error bars in all figures represent the means ± SEM.

To determine whether succinate metabolism affected coagulation, we measured prothrombin time (PT) in H/R rats. H/R induced a profound coagulopathy after 1.5 hours of resuscitation, but DMM treatment upon initiation of resuscitation prevented this effect (Fig. 5A). We also measured several markers of organ injury and dysfunction. We found that H/R caused an increase in plasma lactate, creatinine, and potassium and caused a decrease in plasma glucose. DMM prevented a significant increase in lactate and potassium, reduced the increase in creatinine, and prevented the decrease in glucose (Fig. 5B-F). These results suggest that preventing succinate metabolism reduces acute kidney injury and decreases tissue damage and hypoxia. We also measured survival in the H/R model. We found that without drug intervention, H/R caused greater than 50% mortality after 2 hours of resuscitation. However, DMM prevented deaths across this timeframe (Fig. 5G). Equal levels of hemorrhage in control and DMM-treated animals were confirmed by hematocrit levels (Fig. 5F).

**Fig. 6.**
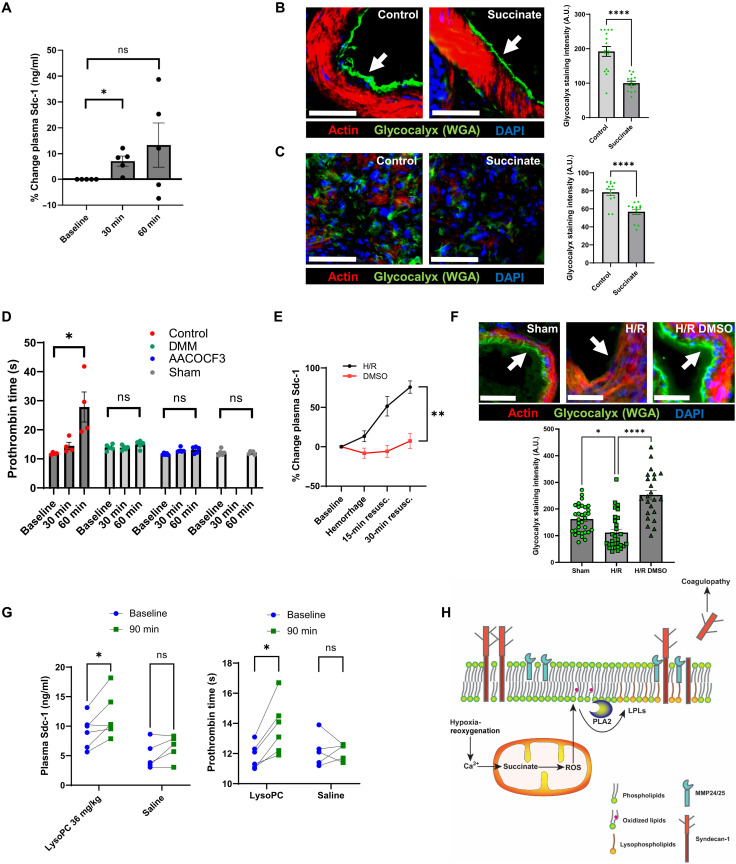
Succinate and lysophospholipid-induced membrane reorganization is sufficient to induce glycocalyx damage and coagulopathy in vivo. (**A**) Increase in plasma syndecan-1 after injection of succinate (1 g/kg). *n* = 5 animals. Significance was assessed with one-way analysis of variance (ANOVA). (**B**) Lung vessel glycocalyx assessed by fluorescent wheat germ agglutinin (WGA) in control or succinate injection (1 g/kg) rats. Actin stain (fluorescent phalloidin) was used for vessel identification. *n* = 16 vessels from four animals per group. Arrows indicate glycocalyx. Scale bars, 20 μm. Significance was assessed with a two-tailed Student’s *t* test. (**C**) Lung alveoli glycocalyx assessed by WGA in control or succinate injection rats. *n* = 13 regions from four animals per group. Scale bars, 20 μm. Significance was assessed with a two-tailed Student’s *t* test. (**D**) Assessment of coagulation using prothrombin time (PT) in succinate injection rats (1000 mg/kg) at baseline and after 30 and 60 min, ± dimethylmalonate (DMM) (50 mg/kg) or AACOCF3 (150 μg/kg). Significance was assessed with two-way repeated measures ANOVA. *n* = 4 to 5 animals per group. Sham control animals were tested at baseline and 60 min. (**E**) Dimethyl sulfoxide (DMSO) intravenous injection (200 μl/kg) prevents glycocalyx shedding as measured by plasma syndecan-1 in hypoxia-reoxygenation (H-R) rats (as in Fig. 4). (**F**) DMSO intravenous injection prevents glycocalyx shedding in tissue sections from lungs in H-R rats. Phalloidin was used for vessel identification. *n* = 12 to 16 vessels from three animals per group. Arrows indicate glycocalyx. Scale bars, 20 μm. Significance was assessed with a two-tailed Student’s *t* test. (**G**) Lysophosphatidylcholine (LPC) intravenous injection induced an increase in plasma syndecan-1 and PT in healthy anesthetized rats after 90 min. Significance was assessed with two-way repeated measures ANOVA. *n* = 5 to 6 animals per group. (**H**) Summary figure of proposed mechanism for acute glycocalyx damage. Error bars in all figures represent the means ± SEM. All ANOVAs were corrected for multiple comparisons using Tukey’s method. A.U., arbitrary units.

We next performed a series of exogenous succinate injection experiments in rats to determine if succinate alone was sufficient to induce glycocalyx damage and coagulopathy. Injection of succinate (1000 mg/kg) intravenously into anesthetized healthy rats induced an increase in plasma syndecan-1 after 30 min (Fig. 6A), and glycocalyx damage was apparent after 60 min in lung tissue sections (Fig. 6, B and C). We also measured PT in these animals and found that succinate injection caused a coagulopathy after 60 min (Fig. 6D). Treatment of the animals with DMM or AACOCF3 before succinate injection prevented the development of coagulopathy (Fig. 6D), suggesting that succinate metabolism and PLA2 activity mediated the effects of exogenous succinate. The succinate was metabolized back to baseline levels after 60 min (fig. S9C).

To assess whether membrane reorganization effects were sufficient to induce glycocalyx damage, we treated H/R rats with intravenous DMSO, which prevented glycocalyx shedding assessed by plasma syndecan-1 levels (Fig. 6E) and lung tissue sections (Fig. 6F). To determine whether LPC elevation is sufficient to induce glycocalyx damage and coagulopathy, we injected LPC intravenously into anesthetized healthy rats and measured plasma syndecan-1 and PT. We found that LPC (36 mg/kg) was sufficient to induce glycocalyx shedding and coagulopathy (Fig. 6G). Our working hypothesis based on cell and animal data is presented in Fig. 6H.

### Trauma plasma samples

**Fig. 7.**
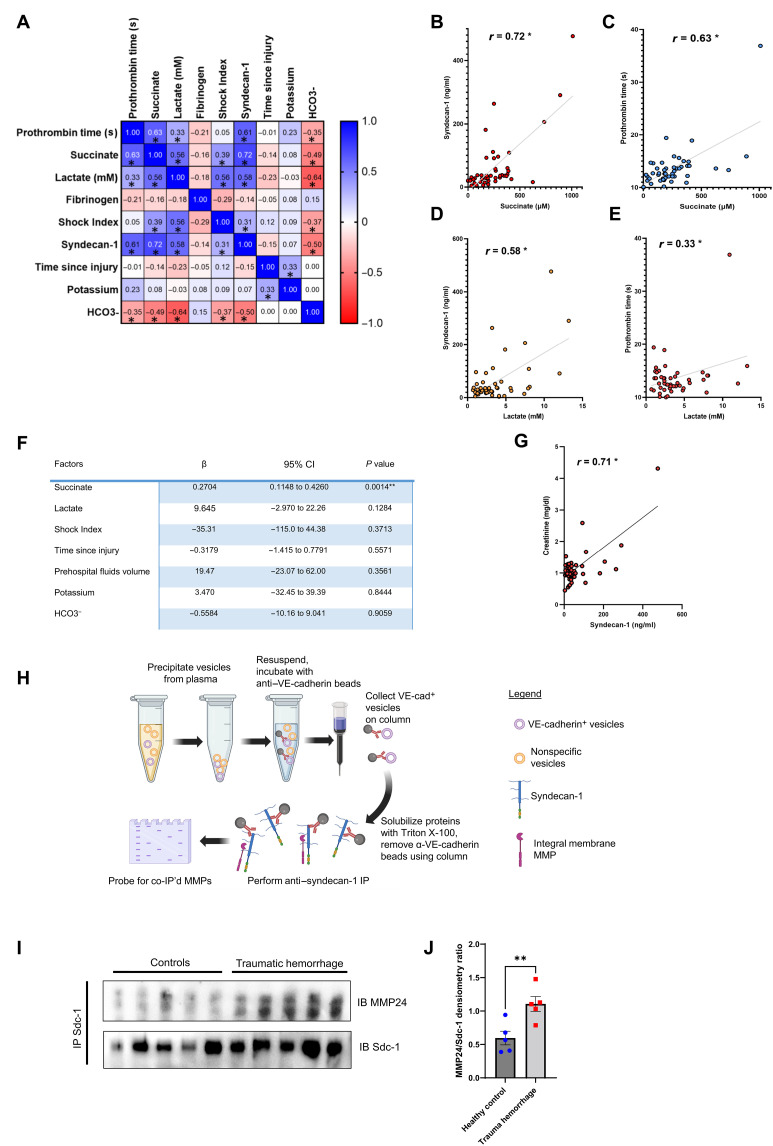
Hemorrhaging trauma patient plasma studies. (**A**) Pearson’s correlation matrix of analyzed variables in the hemorrhaging trauma patient cohort. Significance (*P* < 0.05) marked with an asterisk. (**B**) Correlation between succinate and syndecan-1 levels in plasma from patients with hemorrhaging trauma. (**C**) Correlation between plasma succinate and prothrombin time (PT) in patients with hemorrhaging trauma. (**D**) Correlation between lactate and syndecan-1 levels in plasma from patients with hemorrhaging trauma. (**E**) Correlation between lactate and PT in patients with hemorrhaging trauma. (**F**) Least squares multivariable linear regression analysis on plasma syndecan-1. (**G**) Correlation between creatinine and syndecan-1 levels in plasma from patients with hemorrhaging trauma. (**H**) Protocol flow diagram for isolation of vascular endothelial (VE)–cadherin–positive extracellular vesicles from patient plasma. (**I**) Coimmunoprecipitation (co-IP) of MMP24 and syndecan-1 from VE-cadherin–positive endothelial extracellular vesicles from five healthy controls and five patients with hemorrhage trauma. (**J**) Quantification of co-IP data using densiometry from (H). Significance was assessed with a two-tailed Student’s *t* test. Error bars in figures represent the means ± SEM.

We next determined whether succinate levels were associated with glycocalyx shedding and coagulopathy in bleeding trauma patients. Plasma samples were collected from patients with trauma upon arrival at the emergency department at University Medical Center New Orleans in 2021–2022. Fifty patients with hemorrhaging trauma were included in the study. The demographics of the study population are listed in table S1. Succinate and syndecan-1 levels were measured in the plasma samples, and admission laboratory values were obtained from electronic medical records before deidentification. Figure 7A shows Pearson’s correlation matrix of measured variables from this analysis. The relationship between succinate and syndecan-1 levels is shown in Fig. 7B, and a significant correlation was also observed between succinate and PT (Fig. 7C). The relationship between lactate and syndecan-1 or PT is shown in Fig. 7 (D and E). Succinate levels were more highly correlated with syndecan-1 and PT than lactate levels, supporting the hypothesis that succinate is mechanistically linked with glycocalyx damage and not solely a marker of tissue hypoxia. A least squares multiple linear regression was performed to assess the association between syndecan-1 (dependent variable) and various risk factor–independent variables listed in Fig. 7F. The analysis indicated that succinate was the only independent predictor of syndecan-1 levels in our model (Fig. 7F). We found a significant correlation between syndecan-1 and admission creatinine, suggesting an association between glycocalyx damage and kidney dysfunction in this population (Fig. 7G).

To determine whether the membrane MMPs identified in HUVECs interact to a greater degree with syndecan-1 after hemorrhage, we developed a protocol to isolate endothelial-derived extracellular vesicles from plasma and immunoprecipitate syndecan-1 (Fig. 7H and fig. S10). Although MMP25 was undetectable in these endothelial vesicles, we found higher levels of MMP24 bound to syndecan-1 after hemorrhagic trauma compared to healthy control subjects (Fig. 7, I and J).

To determine whether succinate metabolism may drive glycocalyx damage in other disease states, we performed experiments in HUVECs treated with bacterial lipopolysaccharide (LPS) or H1N1 influenza and established glycocalyx damaging stimuli in our laboratory (*44*, *45*). We found that LPS and H1N1 elevated succinate in HUVECs (fig. S11, A and B). We then measured general glycocalyx levels with WGA and found that the glycocalyx damage induced by LPS and H1N1 could be prevented by DMM. This suggests that succinate metabolism may be a general mediator of glycocalyx damage in a wide range of critical illness states.

## DISCUSSION

Damage to the EG has been recently reported in a variety of critical illnesses, including hemorrhagic and septic shock (*6*, *10*), major surgery (*46*), acute lung injury (*47*), viral infection (*15*), organ transplantation (*48*), myocardial infarction and atherosclerosis (*49*), stroke (*50*), acute renal injury (*51*), and diabetic nephropathy (*52*). This damage is thought to contribute to coagulopathy, dysfunction of tissue blood distribution, edema, and excessive inflammation. These combined effects are now hypothesized to be an important driver of organ dysfunction and death in these critically ill patients (*11*). The mechanism of glycocalyx damage appears to be the up-regulation of a variety of sheddase enzymes in more chronic contexts (*53*). However, glycocalyx damage is apparent after hemorrhage on short time scales and thus must also be mediated by nontranscriptional mechanisms.

We previously reported that in vitro glycocalyx damage could be induced by H-R of cultured endothelial cells, and that mitoROS and Ca^2+^ signaling played a role (*20*). Here, we report that succinate metabolism is the initiating factor that leads to EG degradation in acute hemorrhage. The mechanism involves mitoROS, membrane lipid alterations, and membrane domain rearrangement leading to increased interaction of integral membrane sheddase enzymes with the glycocalyx constituents (Fig. 6H). This work suggests a mechanism for the previously noted association between succinate levels and mortality in critically injured patients (*24*). A recent study suggests an association between glycocalyx shedding and plasma succinate levels which agrees with our findings (*54*).

Succinate has generated increasing attention as a signaling hub linking alterations in mitochondrial metabolism to extramitochondrial effects (*55*). For instance, macrophages respond to lipopolysaccharide by increasing succinate levels, driving ROS production which promotes the expression of pro-inflammatory genes (*56*). This work now demonstrates that succinate can drive mitoROS in endothelial cells as well. This uncovers a pathological mechanism that may play an important role in a wide range of ischemia-reperfusion–related disease states.

This work establishes evidence that ischemia-reperfusion insult to endothelial cells induces an alteration in membrane lipid composition and membrane organization. This leads to an increase in colocalization and molecular interaction between the enzymes MMP24 and MMP25 with syndecan-1. This work suggests a mechanism for the previously noted association between altered lipid metabolism measured at admission and mortality or endotheliopathy in patients with trauma (*54*, *57*). This previously unknown effect on membrane organization could have implications beyond glycocalyx damage. In traumatic brain injury, an alteration in lipid metabolism induced by PLA2 has been implicated in altered endothelial plasma membrane ion channel function (*58*) leading to vascular dysfunction.

Although H-R was sufficient to induce Ca^2+^ signaling leading to succinate elevations and glycocalyx damage in vitro, it is likely that other Ca^2+^-elevating stimuli contribute to the initiation of this succinate-ROS-membrane reorganization pathway in vivo. One likely possibility is that free histones contribute, through their stimulation of endothelial Ca^2+^ elevations (*59*). Catecholamine levels could also promote this pathway, as we observed that, in HPAECs, norepinephrine was required along with H-R to induce glycocalyx damage (fig. S1G).

The coagulopathy of trauma is thought to be due to both autoheparinization of the blood, as well as a consumptive coagulopathy due to activation of the coagulation cascade on the unprotected endothelial surface (*3*, *4*, *11*, *12*). Our results suggest that a notable reordering of the integral proteins of the endothelial membrane, as well as the lipid constituents of this membrane, results from the ischemia-reperfusion insult of H/R. This opens up the possibility that not only is the glycocalyx shed, exposing unprotected endothelial surface but also potentially this reorganization could, in addition, lead to a more pro-coagulant surface than native endothelial membrane. Lipid constituents of platelets are altered during platelet activation to promote coagulation (*60*), and perhaps a similar effect occurs with endothelial lipid alterations. This could suggest that a consumptive coagulopathy on the endothelial surface may play a larger role in the coagulopathy of trauma than autoheparinization. Future work will be needed to investigate this possibility.

In the current work, we used increases in succinyl-lysine as a marker of the cellular location of succinate elevations (Fig. 4F). This was done because of the difficulty in rapidly isolating sufficient endothelial cells to perform a succinate assay. We found succinyl-lysine to be elevated in endothelial cells and in the vascular lumen, this second finding is likely due to elevated plasma succinate. However, this raises the question of whether the succinylation of certain target proteins may drive some of the observed effects of succinate in hemorrhage. Opposing this possibility is the finding that succinate injection in rats mediated coagulopathy through SDH (Fig. 6D). If succinylation was the driver of coagulopathy, then SDH would not be expected to be necessary for this effect, as succinylation is an enzyme-independent reaction (*42*). This reasoning also argues against the succinate receptor, a G protein–coupled receptor expressed in many tissues (*55*), as mediating the effects of succinate in glycocalyx damage.

This work establishes that endothelial-derived extracellular vesicles can be isolated from patient plasma to investigate membrane organization. This relatively simple protocol can be adapted by other investigators to study endothelial membrane biology in a clinically relevant fashion. The relatively small amount of plasma needed (300 μl) should ensure its feasibility in standard clinical and translational sampling protocols.

The use of cultured endothelial cells for the study of the glycocalyx is not universally accepted. Static flow conditions are also suspected to reduce glycocalyx formation (*61*). We have previously shown that the culture of HUVECs in flow conditions did not alter glycocalyx measurements or shedding during H-R insults using our culture techniques (*20*). Careful measurements of glycocalyx by rapid freezing followed by freeze substitution electron microscopy demonstrated an extensive glycocalyx under static cultured conditions (*62*). We and others have found more robust glycocalyx staining when including albumin in the media to support glycocalyx growth, and when fixation of the glycocalyx is performed without first washing cells (*20*, *62*). We propose that static-cultured endothelial cells express a substantial glycocalyx when cultured with sufficient albumin, but washing the cells with albumin-free buffers before fixation destroys this structure. Measurement of the notable glycocalyx in cultured endothelial cells by atomic force microscopy (without fixation or washing) supports this model (*63*). Thus, in the current manuscript, endothelial cells were cultured with 1% bovine serum albumin (BSA) in the media, and fixation was performed by the addition of 10× concentrated formaldehyde (37% by volume) directly to the culture media without an initial wash step. To offset the modest pH lowering effect of BSA, media were treated with concentrated NaOH to return pH to 7.4 before application to cells.

To further assess whether the HUVECs under these conditions express functional glycocalyx, as well as to determine whether syndecan-1 staining corresponds with this functional glycocalyx, we incubated HUVECs with recombinant coagulation FVII and l-selectin, which are expected to be excluded by the glycocalyx to prevent activation of the coagulation cascade and immune cell binding at the endothelial-blood interface. We found that FVII and l-selectin bound to HUVECs almost exclusively in regions without substantial syndecan-1 expression (Fig. 1F and fig. S3B), suggesting that these regions do represent functional glycocalyx. We found that AT III binding was also excluded from syndecan-1 regions (fig. S3C), which seems to contradict the established binding and sequestration of AT III by the glycocalyx (*64*). However, evidence exists that syndecan-4 may be the primary binding partner of AT III (*65*). We found that AT III does notably colocalize with syndecan-4 in HUVECs (fig. S4A). We then costained cells for syndecan-1 and syndecan-4 and found only partial colocalization of the two proteins (fig. S4C). This suggests that thinking of the glycocalyx as a homogeneous structure may be simplistic, and that regional variations likely exist across the cell surface. Future work will be needed in this area.

As in the cultured cell experiments, we are careful to keep the glycocalyx supported by albumin in our other experiments as well. The isolated pulmonary arteries (fig. S8D) were dissected and cannulated in a physiological saline solution (PSS) containing 1% BSA. Tissue samples for sectioning were flash-frozen en bloc without removal or flushing of vascular contents. These frozen samples were cryo-sectioned directly onto positively charged glass slides without fixation, thus preserving the glycocalyx three-dimensional structure supported by vascular luminal contents. Only once these sections had adhered to the slides was fixation performed. We found that methanol fixation of these tissue sections that were adhered to slides preserved the glycocalyx to a greater degree than formalin fixation. Using this technique, we previously reported glycocalyx thickness measurements from 0.7 to 1.4 μm, depending on the organ bed imaged (*21*). These values are similar to those measured by intravital microscopy (*66*–*70*), suggesting that our technique accurately preserves glycocalyx structure.

Induction of coagulopathy in rodents due to trauma or hemorrhage is not universally observed. It is likely that minor differences in experimental protocols determine whether coagulopathy occurs (*71*, *72*). In our model, coagulopathy was not observed until 1.5 hours of resuscitation, and only with severe hemorrhage which reduced the measured hematocrit to 20 to 21%. However, this was not a dilutional coagulopathy, as DMM was able to reverse it without altering hematocrit or resuscitation volume. We used PT as our measure of coagulopathy, as this is the measurement taken for all patients with trauma upon arrival at the emergency department and it is an established measure in rats (*72*). We did not perform rotational thromboelastography, as this has been reported to primarily measure platelet function in rats (*72*–*74*), and glycocalyx shedding is unlikely to induce a primarily platelet-driven coagulopathy.

Persistent damage to the EG, likely due to both ongoing shedding as well as transcriptional down-regulation of glycocalyx components, is reported in trauma and other critical illness states (*45*, *75*–*78*). An increase in succinate metabolism has been reported to drive transcriptional alterations (*31*, *56*) in several cell types. This linkage of succinate-induced mitoROS with transcriptional changes suggests that the mechanism we describe here, which mediates acute glycocalyx damage, could potentially cascade into longer-term, transcriptionally mediated effects as well. Transcriptional changes in trauma have been linked to c-Jun activity (*75*), and succinate has been shown to stabilize HIF-1α (*31*, *56*). As HIF-1α and c-Jun have been shown to cooperatively regulate transcription (*79*), these factors may act as the interface between short-term and long-term endotheliopathy. The succinylation of histones to alter chromatin dynamics may be another possible mechanism of transcriptional alterations (*80*). Future work will be needed to investigate these possibilities.

The results presented in this work suggest that targeting succinate metabolism or endothelial membrane organization may be therapeutic options for endotheliopathy following hemorrhage. We also found that lipopolysaccharide or influenza increases succinate in endothelial cells and that DMM prevents glycocalyx damage from these agents (fig. S11). This suggests that succinate metabolism may be a general mechanism for glycocalyx damage in a wide range of disease states.

We found that blocking succinate metabolism not only corrected the coagulopathy induced by hemorrhage but also improved kidney function and reduced pathological alterations in potassium and glucose. This suggests that either glycocalyx damage contributes to these alterations or blocking succinate metabolism has additional beneficial effects on organ and tissue damage during hemorrhage. These results argue for the development of succinate- or membrane organization–based therapeutics for hemorrhaging and other critically ill patients.

## MATERIALS AND METHODS

### Cell culture

HUVECs and HPAECs were purchased from the American Type Culture Collection. HDMECs were purchased from PromoCell. HUVECs were pooled from 10 donors, both male and female. HPAECs and HDMECs were from single donor. Cells were initially grown in 2% gelatin-coated 10-cm plastic dishes using M200 medium supplemented with low serum growth supplement (LSGS; for HUVECs and HPAECs) or MCDB 131 medium with microvascular growth supplement (Thermo Fisher Scientific), with added penicillin/streptomycin in a cell culture incubator at 37°C with 5% CO_2_ atmosphere. Cells were passaged by digestion in 0.25% trypsin in Hanks’ balanced salt solution after reaching 80% confluence. Cells were used for experiments between passages 1 and 3. For glycocalyx quantification, cells were plated in 96-well plastic cell culture plates coated with 2% gelatin, at a confluence of approximately 90 to 100%. M200 + LSGS (or MCDB 131 + MVGS for HDMECs) + penicillin/streptomycin were supplemented with 1% BSA to support glycocalyx growth (*62*). Media were treated with concentrated NaOH to return pH to 7.4 after the addition of BSA, to offset the acidic nature of BSA. Cells were cultured for 24 hours to allow glycocalyx development before hypoxia exposure. Cells were cultured in the absence of flow conditions. We have previously shown that culture under flow conditions does not alter glycocalyx production or shedding in our model (*20*).

### Immunofluorescence

To investigate the effects of ischemia-reperfusion on the EG, we exposed cultured endothelial cells to a H-R protocol as previously described (*20*). Briefly, cells were moved to a hypoxic chamber (Biospherix) set at 2% O_2_ and 5% CO_2_ for 30 min. For reoxygenation, cells were moved back to a standard cell culture incubator for an additional 30 min. After completion of the hypoxia exposure ± the reoxygenation phase, cells were fixed by the addition of concentrated formaldehyde solution directly to the culture medium to yield a final formaldehyde concentration of 4%. After 10 min of fixation, cells were washed with phosphate-buffered saline (PBS) supplemented with 1% BSA. Cells were then blocked in 1% BSA in PBS for 1 hour (without a permeabilization step, to reduce intracellular labeling). Cells were then incubated with primary antibody overnight in 1% BSA in PBS at 4°C, or fluorescently labeled WGA (1:500) in 1% BSA in PBS at 22°C for 45 min. After three washes with 1% BSA in PBS, cells were incubated with a secondary antibody in % BSA in PBS for 1 hour at room temperature. After three more washes with % BSA in PBS, cells were covered with Fluoro-Gel mounting medium and imaged on an Olympus BX51 fluorescence microscope. Fluorescence staining intensity was quantified using ImageJ software. For DMS, succinate, or LPC (Sigma-Aldrich, L0906) addition experiments, these reagents were added directly to the culture media at the indicated concentrations. Antibodies used throughout the study are listed in Table 1. In the manuscript, WGA staining was performed to measure the effects on total glycocalyx. Syndecan-1 staining was used when effects on total glycocalyx were already established by prior work, and more detailed and higher-resolution images were desired.

**Table 1.** Antibody information.

Name	Product ID	Dilution
Anti-CD31 antibody	Novus Biologicals NB100-2284 RRID:AB_10002513	WB: 1:1000
Anti-eNOS antibody	Cell Signaling #32027 RRID:AB_2728756	WB: 1:1000
Anti-integrin α-IIB	Cell Signaling #13807 RRID:AB_2747364	WB: 1:1000
Anti-CD3ε	Novus Biologicals NBP2-44880 RRID:AB_2923168	WB: 1:1000
Anti–flotillin-1	Cell Signaling #18634 RRID:AB_2773040	WB: 1:1000
Anti-Alix	Cell Signaling #2171 RRID:AB_2299455	WB: 1:1000
Anti-CD9	Cell Signaling #13174 RRID:AB_2798139	WB: 1:1000
Anti–annexin V	Cell Signaling #8555 RRID:AB_10950499	WB: 1:1000
Anti-GM130	Cell Signaling #12480 RRID:AB_2797933	WB: 1:1000
Anti–Syndecan-1	Cell Signaling #12922 RRID:AB_2798062	WB: 1:1000 IP: 1:500
Anti-MMP24	Invitrogen PA1-25244 RRID:AB_794929	WB: 1:1000 IF: 1:100
Anti-MMP25	Invitrogen PA5-76690 RRID:AB_2720417	WB: 1:1000 IF: 1:100
Anti-PLA2	Cell Signaling #2832 RRID:AB_2164442	WB: 1:1000
Anti–Phospho-PLA2	Cell Signaling #2831 RRID:AB_2164445	WB: 1:1000
Anti-MMP16	Invitrogen 701306 RRID:AB_2532467	WB: 1:1000
Anti-MMP17	Invitrogen PA1-32162 RRID:AB_2145648	WB: 1:1000
Anti-MMP14	Cell Signaling #13130 RRID:AB_2798127	WB: 1:1000
Anti-MMP26	ProteinTech 18087-1-AP RRID:AB_2878495	WB: 1:1000
Anti–succinyl-lysine	PTM Bio #PTM-401 RRID:AB_2687628	WB: 1:1000 IF: 1:100
Anti-SIRT5	Thermo Fisher Scientific #TA503232 RRID:AB_11126262	IF: 1:100
Anti–6x-His tag	Thermo Fisher Scientific #4A12E4 RRID: AB_2533309	IF: 1:100
Anti-mouse HRP	Thermo Fisher Scientific #31430 RRID:AB_228307	WB: 1:5000
Anti-rabbit HRP	Thermo Fisher Scientific #31460 RRID:AB_228341	WB: 1:5000
Anti-rabbit light chain HRP	Millipore #MAB201P RRID:AB_827270	WB: 1:5000
Anti-rabbit Alexa Fluor 568	Invitrogen A-11011 RRID:AB_143157	IF: 1:500
Anti-mouse Alexa Fluor 555	Invitrogen A32727 RRID:AB_2633276	IF: 1:500
Anti-rabbit Alexa Fluor 488	Invitrogen A32790TR RRID:AB_2866495	IF: 1:500
Anti-mouse Alexa Fluor 594	Thermo Fisher Scientific #A21203 RRID:AB_141633	IF: 1:500
Anti-rabbit Alexa Fluor 594	Thermo Fisher Scientific #A-21207 RRID: AB_141637	IF: 1:500
Anti-goat Alexa Fluor 488	Thermo Fisher Scientific #A-11055 RRID: AB_2534102	IF: 1:500

### Factor VII, l-selectin, and antithrombin III binding

Recombinant His-tagged human FVII and recombinant His-tagged AT III were purchased from R&D Systems (2338-SE, 1267-PI-010). Recombinant His-tagged l-selectin was purchased from Thermo Fisher Scientific (RP-75647). Cells were exposed to normoxia or H-R as above. Recombinant proteins were added to wells at a final concentration of 2 nM immediately before the normoxia or the H-R protocol, such that the proteins were in contact with cells for 1 hour for both treatment conditions. Cells were fixed, blocked, and stained as above, using anti–syndecan-1 and anti–His tag antibodies. Colocalization analysis was performed using ImageJ (JACoP plugin).

### Mitochondrial ROS, succinate measurements, TBARS assay.

MitoSOX was used as an indicator of mitoROS levels. mitoSOX was dissolved in DMSO and added to cells at 1:5000 dilution, at a final concentration of 1 μM. H-R or succinate addition studies were then performed as above.

Succinate was measured in plasma and HUVEC lysates using a commercially available kit (LSbio). TBAR assay was performed on HUVEC lysates as indicated by the manufacturer (Cayman Chemical) and controlled to a level of total protein per sample, as measured by Bradford assay.

### Immunoprecipitation and SDS-PAGE

HUVECs were lysed in lysis buffer [50 mM tris-HCl (pH 7.5), 150 mM NaCl, 0.5 M EDTA, 1% Triton X-100, and Halt Protease Inhibitor Cocktail (Thermo Fisher Scientific, Waltham, MA)]. Volume was expanded to 1 ml using the lysis buffer and preclearance was performed using immunoglobulin G (20 μl) and protein A magnetic beads (20 μl of 50% slurry). Beads were removed by a magnet, and then primary antibody was added to lysate for 3 hours (anti–syndecan-1, 30 μl). Protein complexes were precipitated using protein A magnetic beads (25 μl 50% slurry) for 1 hour at 4°C. Beads were collected by a magnet and washed three times using PBS. Protein was then released from beads using 30 μl of loading buffer [62.5 mM tris-HCl (pH 6.8), 2.5% SDS, 5% β-mercaptoethanol, and 10% glycerol) boiled for 10 min. Fifteen microliters of each sample was SDS-PAGE on a 10% acrylamide gel run at 100 V. Proteins were then transferred to 0.45-μm polyvinylidene difluoride membrane at 30 V for 2 hours. Membranes were blocked in tris-buffered saline (137 mM NaCl and 20 mM tris base), 0.1% Tween 20, and 5% BSA (blocking solution) for 1 hour, followed by overnight incubation with primary antibody diluted in tris-buffered saline, 0.1% Tween 20, and 3% BSA, and 1-hour incubation with horseradish peroxidase–conjugated secondary antibody (light chain only) diluted at 1:5000. Antibodies used were the following: Rabbit anti–syndecan-1, JM11-21, Novus Biologicals; rabbit anti MMP14, D1E4, Cell Signaling; rabbit anti-MMP16, 13H7L7, Invitrogen; rabbit anti-MMP17, PA1-32162, Invitrogen; rabbit anti MMP24, PA1-25244, Invitrogen; rabbit anti MMP25, PA5-76690, Invitrogen; rabbit anti MMP26, 18087-1-AP, Proteintech.

### Ca^2+^ imaging

HUVECs were loaded with Fluo-4 AM (10 μM) with pluronic acid (0.25%) for 45 min at 37°C in plastic 48-well culture plates before imaging. Cells were then washed with M200 media without phenol red and placed into the Cytation5 imager. O_2_ was reduced to 2% by injection of N_2_, and images were acquired every 90 s for 30 min at 488-nm fluorescence wavelength. Chamber was then returned to normoxia, and cells were further imaged for 30 min. Five images before the start of hypoxia were used to establish baseline values, and the change in fluorescence from this baseline value (*F*/*F*_0_) was calculated for all images.

### Protease assays

For the BAPNA assay, a stock solution (0.1 mg/ml) was made in Hepes-buffered PSS [1.2 mM NaH_2_PO_4_, 140.7 mM NaCl, 2.8 mM CaCl_2_, 1.7 mM MgCl_2_, 10 mM Hepes, 5 mM glucose, 0.5 mM EDTA, and 5.8 mM KCl (pH 7.4)]. Media were removed from HUVECs grown in 96-well plates and replaced with the BAPNA/Hepes solution. Baseline absorbance was measured at 405 nm, and then cells were subjected to hypoxia/reoxygenation protocol. Absorbance at 405 nm was measured again, and the change in absorbance was analyzed as a measure of protease activity. For the casein assay, we used a commercial kit (Pierce Protease Assay Kit, Thermo Fisher Scientific) as per the manufacturer’s instructions. Briefly, we dissolved 10 mg of succinylated casein into 5 ml of Hepes-buffered PSS. A total of 250 μl of this solution was added to wells of 24-well plates containing HUVECs. Cells were subjected to the H-R protocol, and media were collected. Media were centrifuged at 1000*g* to remove cell debris, and the assay was completed using this solution as per the manufacturer’s instructions. For fluorescent quenched gelatin assay (DQ gelatin, Invitrogen), 100 μg/ml of the substrate was diluted in Hepes-buffered PSS and added to 96-well plates containing HUVECs. Cells were subjected to the H-R protocol, and fluorescence changes (488 nm) were recorded over this time frame as a measure of specific protease activities. For the MMP7 activity, we used a commercial kit (Enzo Life Sciences) as per the manufacturer’s instructions. For MMP14, ADAM17, and MMP3 activity, we used commercially available kits (AnaSpec) as per the manufacturer’s instructions. Gelatin zymography of culture media after normoxia or H-R was performed as previously described (*81*). Briefly, media were concentrated 10-fold using Amicon Ultra-0.5 Centrifugal Filters (Millipore Sigma). Media were then loaded into gelatin-containing PAGE gel using a nondenaturing loading buffer. SDS was removed using wash buffers. Gel was incubated overnight at 37°C. Coomassie blue stain was used to image negative bands in gel, indicating MMP gelatinase activity.

### Metabolomics analysis

HUVECs were grown and treated for normoxia or H-R as above. Cells were scraped and homogenized by pipette in ice-cold PBS. Lysates were collected and frozen for later analysis. Samples were thawed and added with 800 μl of 80% methanol, vortexed for 30 s. Then, all samples were extracted at 4°C with ultrasound for 30 min, kept at −40°C for 1 hour, and centrifuged at 12,000 rpm and 4°C for 15 min. Last, the supernatant was dried down under nitrogen gas and the residue was resuspended with 100 μl of 80% methanol and 5 μl of dl-o-chlorophenylalanine (140 μg/ml) for liquid chromatography–mass spectrometry (LC-MS) analysis. Ultra-performance LC–time-of-flight–MS separation was performed by Ultimate 3000LC combined with Q Exactive MS (Thermo Fisher Scientific) and screened with electrospray ionization MS (ESI-MS). The LC system is composed of an ACQUITY UPLC HSS T3 (100 × 2.1 mm 1.8 μm) with Ultimate 3000LC. The mobile phase is composed of solvent A (0.05% formic acid-water) and solvent B (acetonitrile) with gradient elution (0 to 1 min, 5% B; 1 to 12 min, 5 to 95% B; 12 to 13.5 min, 95% B; 13.5 to 13.6 min, 95 to 5% B; 13.6 to 16.0 min, 5% B). The flow rate of the mobile phase is 0.3 ml·min. The column temperature is maintained at 40°C, and the sample manager temperature is set at 4°C. MS parameters in ESI^+^ and ESI^−^ mode are listed as follows: ESI^+^: Heater Temp, 300°C; Sheath Gas Flow rate, 45 arb; Aux Gas Flow Rate, 15 arb; Sweep Gas Flow Rate, 1 arb; spray voltage, 3.0 kV; Capillary Temp, 350°C; S-Lens RF Level, 30%. ESI^−^: Heater Temp, 300°C; Sheath Gas Flow rate, 45 arb; Aux Gas Flow Rate, 15arb; Sweep Gas Flow Rate, 1 arb; spray voltage, 3.2 kV; Capillary Temp, 350°C; S-Lens RF Level, 60%. The raw data are acquired and aligned using the Compound Discoverer (3.0, Thermo Fisher Scientific) based on the mass/charge ratio value and the retention time of the ion signals. Ions from both ESI^−^ and ESI– are merged and imported into the SIMCA-P program (version 14.1) for multivariate analysis. A principal components analysis is first used as an unsupervised method for data visualization and outlier identification. Supervised regression modeling is then performed on the dataset by use of partial least squares discriminant analysis (PLS-DA) or orthogonal PLS-DA to identify significantly altered metabolites. The biomarkers are filtered and confirmed by combining the results of the variable importance in projection (VIP) values (VIP > 1.5) and *t* test (*P* < 0.05). *Z* scores were calculated by comparing individual replicate metabolite log-transformed median scaled values to the associated mean and SD of the entire set of replicates.

### H1N1 and LPS experiments

HUVECs were cultured as described above. Some cells were exposed to A/PR/8/34 H1N1 (PR8) from a frozen stock propagated in the laboratory at a multiplicity of infection of 5 for 24 hours. Some cells were treated with LPS (1 μg/ml) for 24 hours. Cells were then fixed by direct addition of concentrated formaldehyde to culture media to a final concentration of 3.5%. After 10 min of fixation, cells were washed with PBS containing 1% BSA. Cells were then stained with FITC-WGA (23 μg/ml) and 4′,6-diamidino-2-phenylindole (DAPI; 23 μg/ml) in PBS with 1% BSA for 20 min at room temperature in the dark. Cells were then washed twice with 1% BSA in PBS and covered with Fluoro-Gel mounting medium. Glycocalyx was imaged on an EVOS fluorescence microscope at 488 nm under identical conditions. ImageJ software was used to quantify the glycocalyx staining intensity.

### Animals

All animal care and experimentation were performed in accordance with the Tulane University Institutional Animal Care and Use Committee–approved protocols, and following guidelines from the Institute for Laboratory Animal Research. Study subjects were male Sprague-Dawley rats (Charles River Laboratories) weighing 225 to 350 g and 6 to 10 weeks old. Preliminary studies suggested that female rats required a more severe insult to elicit glycocalyx damage and may be resistant to succinate elevations. A future study will investigate these differences. ARRIVE guidelines were followed in the design and performance of animal studies.

### Hemorrhage procedure

Following procedures originally described in (*21*), anesthesia was induced in a small animal anesthesia chamber with 4% isoflurane in 100% O_2_; anesthesia was maintained throughout the surgical preparation and procedure with 2% isoflurane in O_2_. Anesthesia was delivered to all animals using a nose cone throughout the procedure, and animals were positioned such as to expose the anterior body surface. A right femoral artery cannula was connected to a pressure transducer for monitoring MAP. An additional left femoral artery cannula was used for controlled blood removal and collection. A right external jugular vein cannula was placed for the perfusion of resuscitation fluids and drugs into the animals. Patency was maintained in all cannulations using heparin (200 U/ml) or 1% sodium citrate in PBS. Sodium citrate was exclusively used in place of heparin for all studies where PT was measured. Blood samples were collected from the right femoral cannula. Ten microliters of heparin solution (5000 U/ml) was added to all blood samples to prevent coagulation; samples were stored at 4°C after collection for the duration of the surgical procedure. We performed hemorrhagic shock using a modification of Wiggers’ protocol (*82*). The animal was hemorrhaged by controlled blood removal via the left femoral cannula until a MAP of 40 ± 3 mmHg was achieved. Hemorrhagic shock was maintained for 30 min by removing blood to maintain a MAP of 40 mmHg. Rats were then resuscitated with lactated Ringer’s (LR) solution. LR solution was perfused via the internal jugular vein cannula at ∼2 ml/min until a MAP of ≥60 mmHg was achieved, and then periodically to maintain resuscitation. Drugs (if any) were administered immediately before the resuscitation phase, through the jugular catheter. Drugs were dissolved in 1 to 2 ml of LR solution, which we counted into the total resuscitation volume. The resuscitation period was maintained for 30 min for some experiments and 2 hours for other experiments. Equal levels of hemorrhage in control and DMM-treated rats were confirmed by hematocrit evaluation (Fig. 4F). Euthanasia was performed by severing the diaphragm followed by exsanguination. Tissue samples were immediately collected and embedded in an optimal cutting temperature compound (Tissue-Tek) and snap-frozen in liquid nitrogen. Tissue was preserved at −40°C until samples were collected from all study subjects. Sham rats were anesthetized and all three vascular lines were placed as above, but no blood was removed. PT, lactate, creatinine, potassium, glucose, and hematocrit were measured using an iSTAT portable blood analyzer. The sample size was determined on the basis of prior studies (*21*). Animals were randomized between groups by coin flip, and analysis of plasma and tissue sections was performed under blinded conditions.

### Succinate and lysophosphatidylcholine injection rats

Rats were anesthetized as above. Cannulas were placed in the jugular vein and one femoral artery. Baseline blood samples were taken for coagulation and enzyme-linked immunosorbent assay (ELISA) analysis. Succinate (1000 mg/kg) in PBS or LPC (36 mg/kg) (Sigma-Aldrich, L0906) was administered through the jugular. Thirty and 60 min later for succinate or 90 min later for LPC, additional blood samples were taken. After the 60-min time point for succinate experiments, tissue was collected for the hemorrhage rats. LPC dose was determined using the following estimation: Endogenous LPC levels in rodent plasma are ~400 μM (*83*), ~6 mg/kg.

So to double this amount, we need to inject 6 mg/kg. Estimating that two of three of injected LPC are bound by albumin (*84*) and up to half is taken up by erythrocytes, this gives 6 × 2 × 3 = 36 mg/kg.

### Syndecan-1 ELISA

Collected blood samples were centrifuged for 5 min at 500*g* at 4°C to collect plasma. To quantify solubilized (shed) glycocalyx, we analyzed plasma syndecan-1 levels using an ELISA (Novus Biologicals, NBP2-76611) as per the manufacturer’s instructions.

### Tissue section glycocalyx imaging

Following procedures originally described in (*21*), flash-frozen tissue was then sectioned in a cryostat at −20°C at a section thickness of 10 μm; the sections were affixed to room-temperature glass slides. Fixation was performed by immersion in 4°C methanol for 10 min. Glycocalyx staining was performed with FITC-WGA (1:1000) in PBS for 1 hour at room temperature with DAPI. Some organs were also incubated with Alexa-Fluor 594 phalloidin to aid in identifying the vasculature. Sections were washed three times with PBS and coverslipped in Fluoro-Gel (Electron Microscopy Sciences). Because of the thickness of the tissue sections, syndecan-1 staining did not resolve the spatial organization of the glycocalyx to a greater degree than WGA staining. Therefore, we performed only WGA stains on tissue sections.

Succinyl-lysine and SIRT5 immunofluorescence sections were collected and sectioned as above but were fixed with 4% paraformaldehyde solution in PBS for 10 min at room temperature. Sections were then washed three times with PBS. Sections were blocked with 1% BSA in PBS for 1 hour. Then, sections were incubated with anti–succinyl-lysine antibody (1:100 dilution in 1% BSA in PBS; PTM Biolabs, PTM-401) or anti-SIRT5 antibody (1:100 dilution in 1% BSA in PBS; Thermo Fisher Scientific, #TA503232) overnight at 4°C. Sections were subsequently washed three times with PBS and incubated with anti-rabbit or anti-mouse Alexa Fluor secondary antibody (1:500 dilution in 1% BSA in PBS) with DAPI and phalloidin for 1 hour at room temperature. Sections were then washed three times with PBS and coverslipped using Fluoro-Gel. Tissue was imaged on an Olympus BX51 fluorescence microscope. Glycocalyx, SIRT5, and succinyl-lysine staining intensity were quantified using ImageJ.

### Live vessel glycocalyx imaging

After completion of the H/R protocol, the lungs were quickly dissected out and placed in ice-cold Hepes-buffered PSS [Hepes PSS: 1.2 mM NaH_2_PO_4_, 140.7 mM NaCl, 2.8 mM CaCl_2_, 1.7 mM MgCl_2_, 10 mM Hepes, 5 mM glucose, 0.5 mM EDTA, and 5.8 mM KCl (pH 7.4)] with 1% BSA. Third-order pulmonary arteries were dissected out and placed in fresh Hepes PSS with 1% BSA. Vessels were cannulated on glass cannulas in heated, oxygenated perfusion bath chambers (Living Systems Instrumentation). Arteries were pressurized to 15 mmHg and 100 μl of 1:1000 fluorescent WGA in Hepes PSS with 1% BSA was passed through the lumen. Fluorescence and bright-field images were collected on an Accu-Scope fluorescent stereomicroscope and analyzed using ImageJ.

### Trauma patient plasma samples

Deidentified patient samples were collected with the approval of the Tulane University Institutional Review Board (IRB). Waiver of consent was granted by the IRB because of minimal risk (samples were collected as part of standard care), lack of practicality, and deidentification of samples. Blood samples from patients with trauma were collected upon arrival to the trauma bay in citrated tubes. Inclusion criteria were patients with hemorrhaging trauma 18 years or older, exclusion criteria were visibly or verbally reported pregnant women known prisoners. Data points were not omitted from the analysis. Blood was spun at 750*g* for 10 min at 4°C to collect plasma. Plasma was stored at −40°C until analysis. Plasma succinate assay was performed as above, and syndecan-1 levels were measured by ELISA (Abcam, ab46506) as per the manufacturer’s instructions. Blood chemistry and clinical data were collected from hospital electronic medical records.

### Trauma patient plasma extracellular vesicle experiments

Plasma samples from patients with trauma or healthy controls (300 μl) were mixed with exosome precipitation solution (Novus Biologicals) as per the manufacturer’s instructions. Samples were incubated at 4°C for 30 min with tubes oriented upright. Samples were then centrifuged for 30 min at 1500*g* at 4°C. The supernatant was aspirated, followed by additional centrifugation at 1500*g* and 4°C for 5 min. The supernatant was then aspirated again. Pellet was resuspended in 1000 μl of PBS. One hundred microliters was separated out and frozen for later analysis. One hundred microliters of anti–vascular endothelial (VE)–cadherin magnetic microbeads (Miltenyi Biotec) was added to each sample and incubated for 15 min at 4°C. Samples were then passed through a magnetic column (MS Column, Miltenyi Biotec), discarding flow through. The column was washed three times with 0.1% BSA in PBS. The column was removed from magnet and contents flushed with 1000 μl of PBS to remove retained material. Triton X-100 was then added to this flow through to a final concentration of 0.1%. Samples were vigorously inverted for ~10 s. Fifty microliters of each sample was separated and frozen for later analysis. The remaining material was then subjected to syndecan-1 immunoprecipitation as described above for cell lysates. Particle size was performed using a ZetaView nanoparticle tracking analyzer (Particle Metrix). Diluted samples of eluted vesicles were imaged using a 488-nm laser with no filter (scatter mode) with the following parameters: Minimum brightness 30, max area 1000, minimum area 10, trace length 15, nm/Class 10, sensitivity 80.0, frame rate 30.0, shutter 100, temperature 25.0. Eleven positions were imaged per sample.

### Figure conventions used in this manuscript

All figures in this manuscript use the *P* value symbol convention as follows: ns, *P* > 0.05; *P ≤ 0.05, **P ≤ 0.01, ***P ≤ 0.001, and ****P ≤ 0.0001.

## Notes

View/request a protocol for this paper from *Bio-protocol*.
