# Supplementary material for: Succinate metabolism and membrane reorganization drives the endotheliopathy and coagulopathy of traumatic hemorrhage
Source: Sci Adv. 2023 Jun 14;9(24):eadf6600. doi: 10.1126/sciadv.adf6600 (PMC10266735; doi:10.1126/sciadv.adf6600)
Supplement: Supplementary file 1 — Figs. S1 to S11 Table S1 [file sciadv.adf6600_sm.pdf]

Science Advances
AAAS

Supplementary Materials for

Succinate metabolism and membrane reorganization drives the endotheliopathy and coagulopathy of traumatic hemorrhage

Sarah Abdullah *et al.*

Corresponding author: Olan Jackson-Weaver, ojacksonweaver@tulane.edu

Sci. Adv. **9**, eadf6600 (2023)
DOI: 10.1126/sciadv.adf6600

This PDF file includes:

Figs. S1 to S11
Table S1

Supplemental Figures

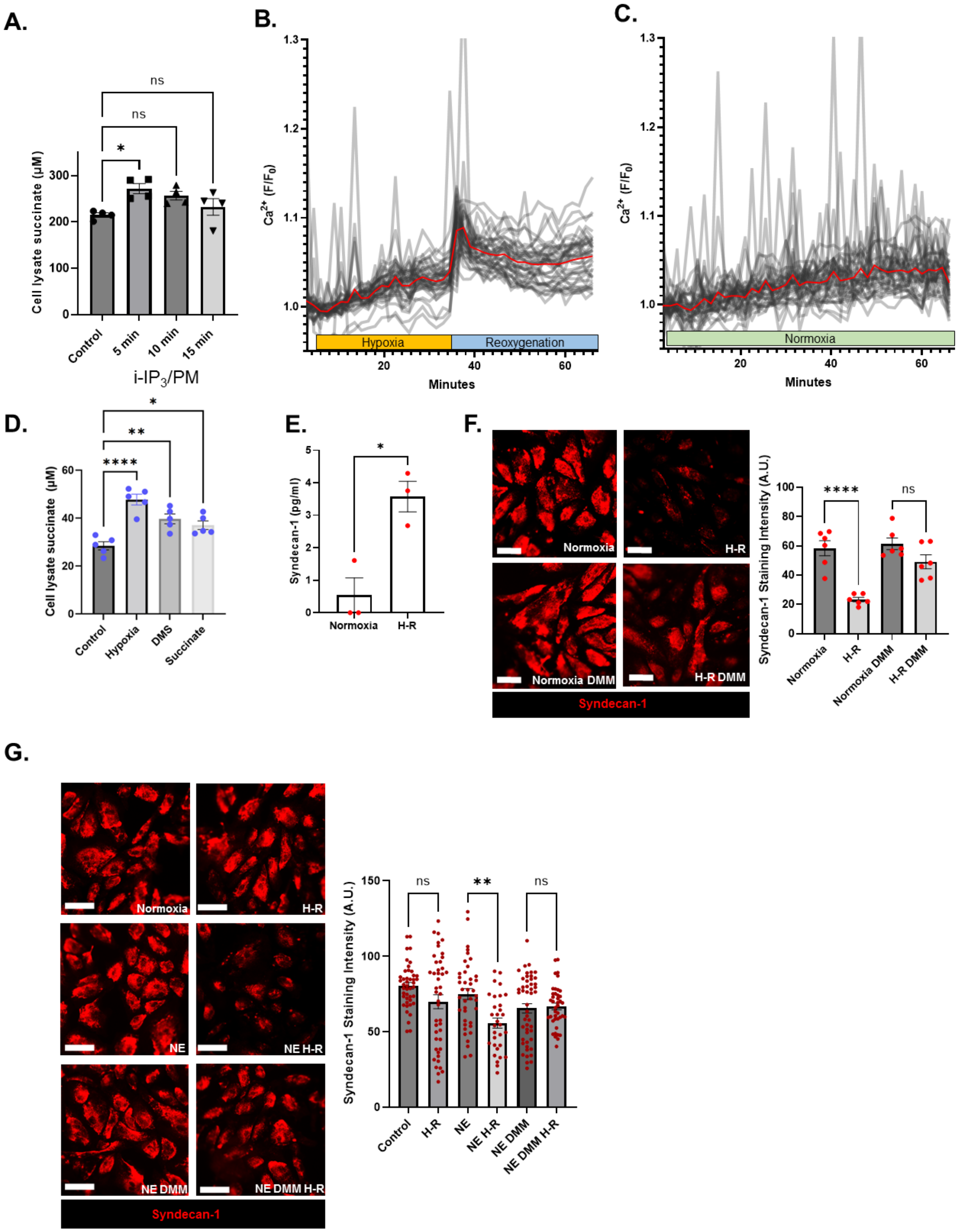

Figure S1

Fig. S1: Succinate mechanisms and measurements in cultured cells.
A. Cell lysate succinate measurements from control or cell permeable IP_3 analog i-IP_3/PM for inticated times. n = 4 biological replicates. Significance was assessed with one-way ANOVA corrected for multiple comparisons using Tukey's method. **B.** Measurement of intracellular Ca^{2+} in HUVECs subjected to hypoxia-reoxygenation protocol. Ca^{2+} traces were measured by Fluo-4 fluorescence from 37 cells, recorded from 3 biological replicate wells. Red trace indicates average. **C.** Time control of Fluo-4 Ca^{2+} measurements. Protocol was identical to experiments in B., but cells were exposed to normoxia only. n=38 cells, recorded from 3 biological replicate wells. Red trace indicates average. **D.** Cell lysate succinate measurements from control (normoxia) or hypoxic (30 minutes, 2% O_2) HUVECs, or control cells treated with dimethylsuccinate (DMS, 50 μM) or succinate (2 mM) for 30 minutes prior to collection. n = 5 biological replicates. Significance was assessed with one-way ANOVA corrected for multiple comparisons using Tukey's method. **E.** Soluble syndecan-1 is elevated in HUVEC media following H-R as assessed by ELISA. n=3 biological replicates. **F.** Human dermal microvascular endothelial cells (HDMECs) exhibit syndecan-1 shedding following H-R protocol. Dimethylmalonate (DMM, μM) prevented this effect. n=6 biological replicates. Scale bars = 60 μm. Significance was assessed with one-way ANOVA corrected for multiple comparisons using Tukey's method. **G.** Human pulmonary artery endothelial cells (HPAECs) exhibit syndecan-1 shedding after H-R in the presence of norepinephrine (NE, 10 μM). This effect was prevented by treatment with dimethylmalonate (DMM μM). Scale bars = 60 μm. Significance was assessed with one-way ANOVA corrected for multiple comparisons using Tukey's method. Error bars in all figures represent the mean ± s.e.m.

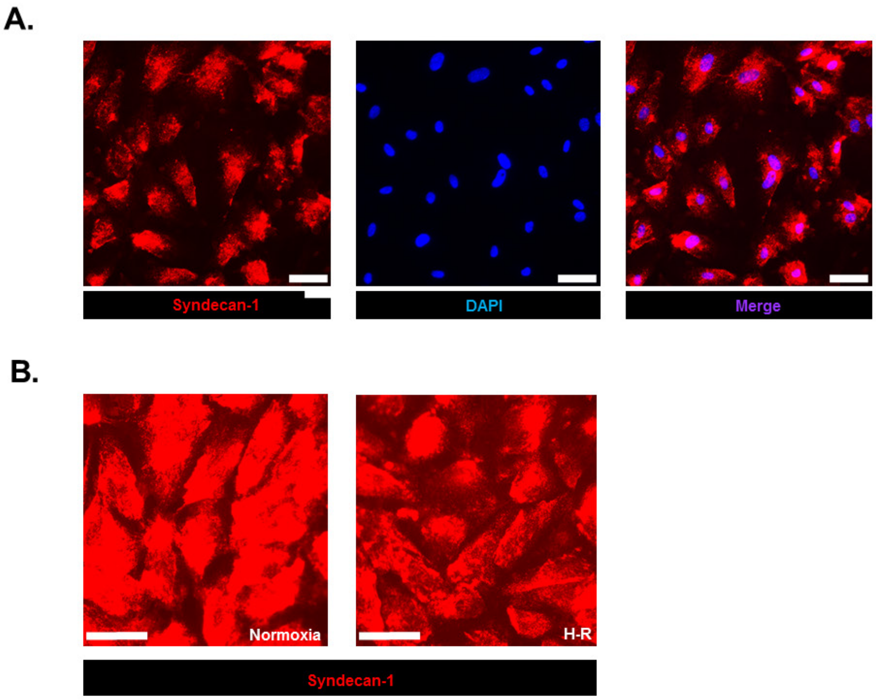

Figure S2

Supplemental Fig. S2: Syndecan-1 distribution in HUVECs. A. High magnification view of syndecan-1 immunostaining in HUVECs. High staining intensity is localized to the center of the cell mass, but is not specifically a nuclear localization, as shown by DAPI overlay. Scale bars = 60 µm. **B.** Longer exposure immunofluorescence image of syndecan-1 in HUVECs exposed to normoxia or H-R demonstrate that cells are 90-100% confluent in both conditions. Scale bars = 60 µm.

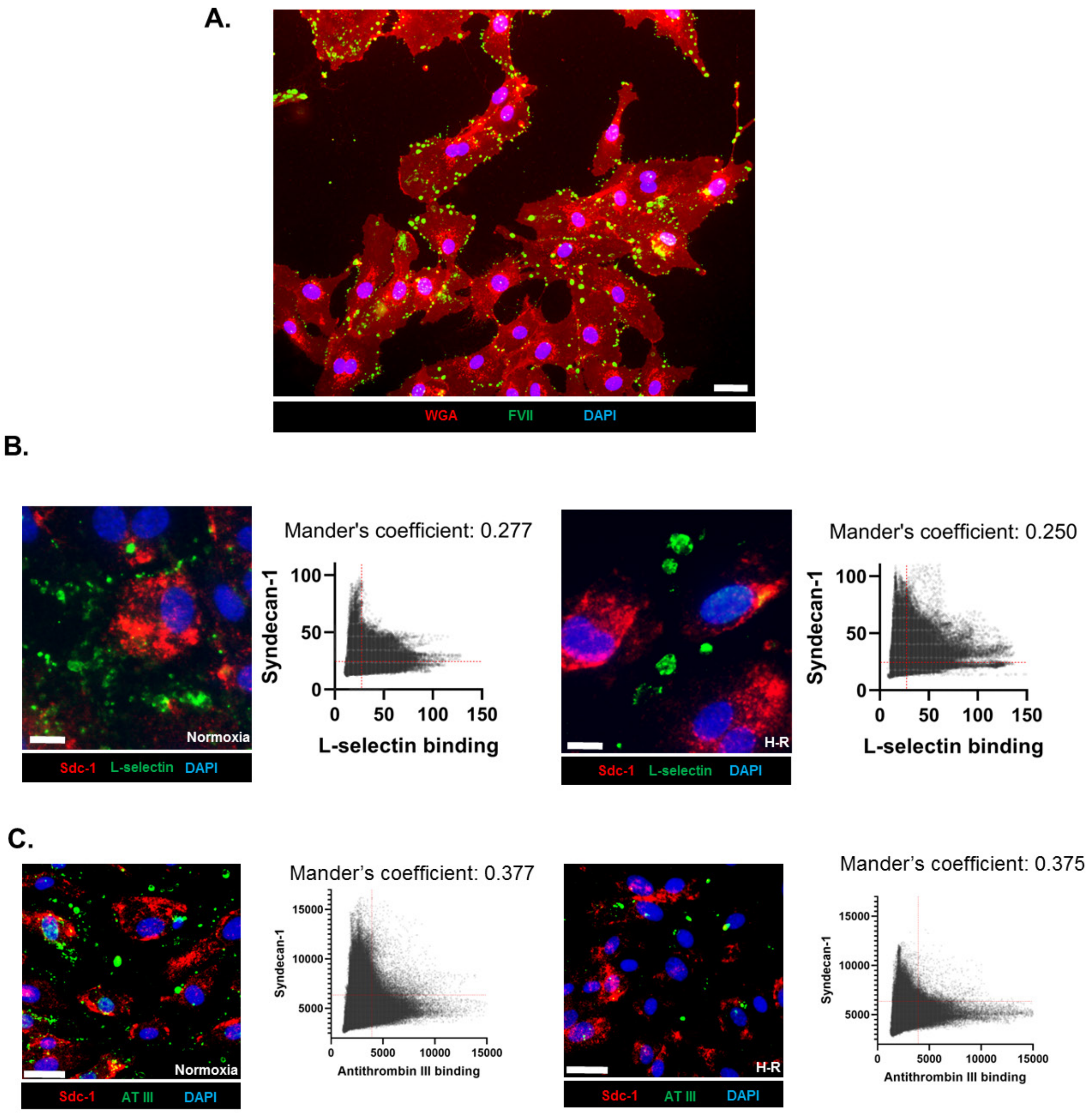
A.
WGA
FVII
DAPI
B.
Normoxia
Sdc-1
L-selectin
DAPI
Mander's coefficient: 0.277
Syndecan-1
L-selectin binding
H-R
Mander's coefficient: 0.250
C.
Normoxia
AT III
Mander's coefficient: 0.377
Antithrombin III binding
Mander's coefficient: 0.375

Figure S3

Supplemental Fig. S3: Recombinant protein binding experiments. A. Recombinant factor VII binds to HUVECs and not to cell-free regions of the well as shown by overlay with wheat germ agglutinin (WGA) staining. Scale bar = 30 µm. **B.** Recombinant human L-selectin is excluded from syndecan-1 regions in cells exposed to normoxia or H-R. Colocalization data is shown in cytofluorograms and quantified by Mander's coefficient. Scale bars = 20 µm. Representative images and cytofluorograms from 3 biological replicates. **C.** Recombinant human antithrombin III is excluded from syndecan-1 regions in HUVECs exposed to normoxia or H-R. Colocalization data is shown in cytofluorograms and quantified by Mander's coefficient. Scale bars = 50 µm. Representative images and cytofluorograms from 3 biological replicates.

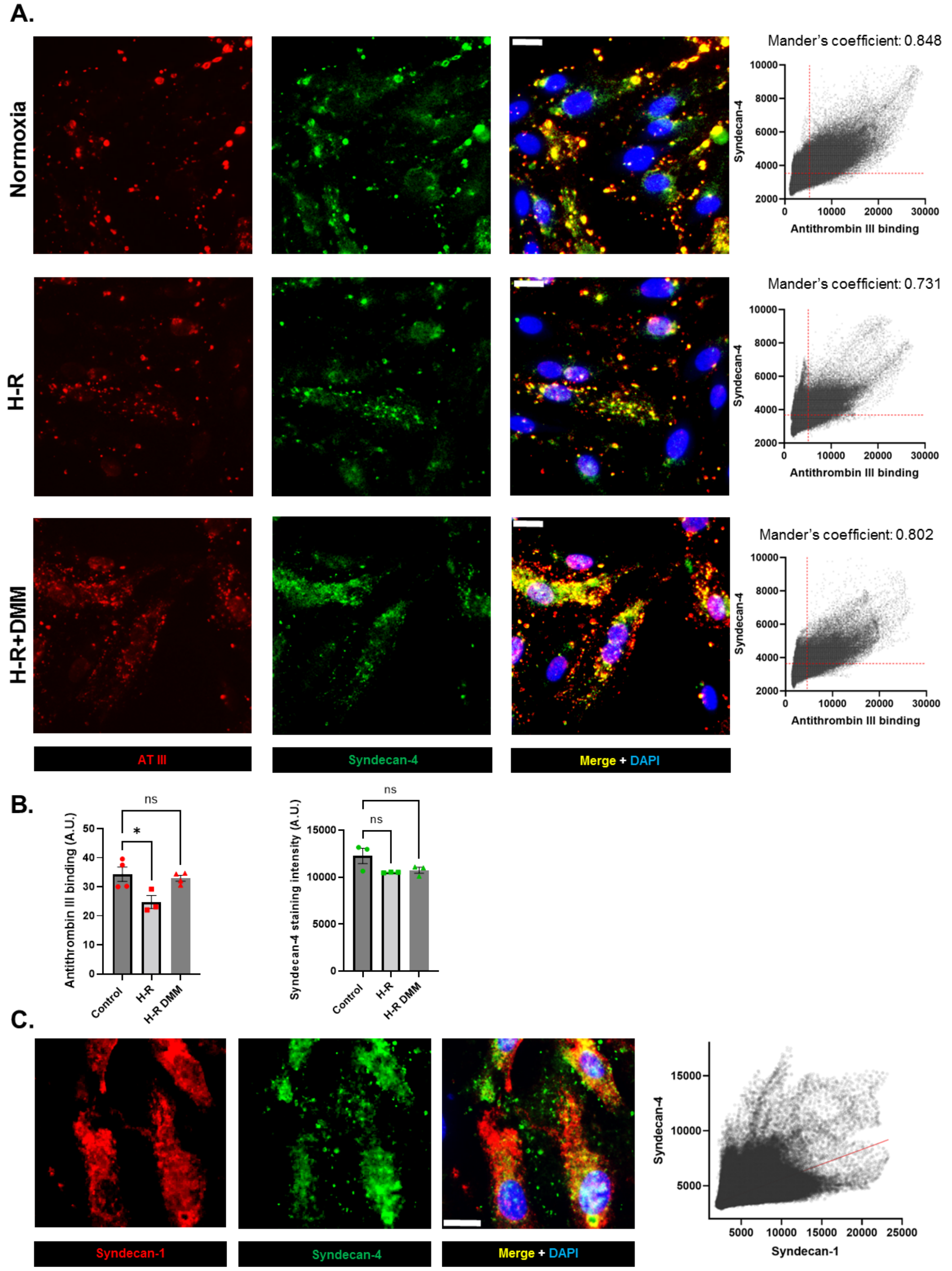
A.
Normoxia
H-R
H-R+DMM
Mander's coefficient: 0.848
Mander's coefficient: 0.731
Mander's coefficient: 0.802
Syndecan-4
Antithrombin III binding
AT III
Syndecan-4
Merge + DAPI
B.
Antithrombin III binding (A.U.)
Syndecan-4 staining intensity (A.U.)
ns
*
Control
H-R
H-R DMM
C.
Syndecan-1
Syndecan-4
Merge + DAPI

Figure S4

Supplemental Fig. S4: Antithrombin III binds to syndecan-4 domains. A. Recombinant antithrombin III binding to HUVECs co-localizes with syndecan-4 under normoxia, H-R, or H-R treated with dimethylmalonate (DMM, 50 µM) conditions. Cytofluorograms illustrate linear co-localization, and this is quantified with Mander's coefficient. Representative images and cytofluorograms from 3 biological replicates. Scale bars = 20 µm. **B.** Quantification of antithrombin III binding and syndecan-4 levels in HUVECs during normoxia, H-R, or H-R treated with dimethylmalonate (DMM, 50 µM). Significance was assessed with one-way ANOVA corrected for multiple comparisons using Tukey's method. n=3-4 biological replicates. **C.** Co-immunofluorescence of syndecan-1 and syndecan-4 demonstrate only partial co-localization, with punctate staining of syndecan-4 outside of syndecan-1 regions. Cytofluorogram illustrates complex spatial relationship. Representative images and cytofluorogram from 3 biological replicates. Scale bar = 20 µm.

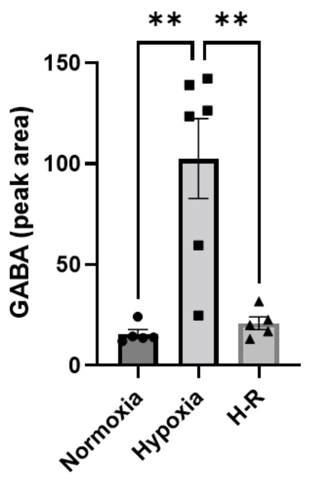

Figure S5

Supplemental Fig S5: GABA induced by hypoxia in endothelial cells.

ɣ-aminobutyric acid (GABA) levels as assessed by metabolomics analysis in HUVECs subjected to normoxia, 30 minutes of hypoxia (30 minutes, 2% O_2), or hypoxia-reoxygenation (H-R) protocol. n = 6 biological replicates. Significance was assessed with one-way ANOVA corrected for multiple comparisons using Tukey's method. Error bars in all figures represent the mean ± s.e.m.

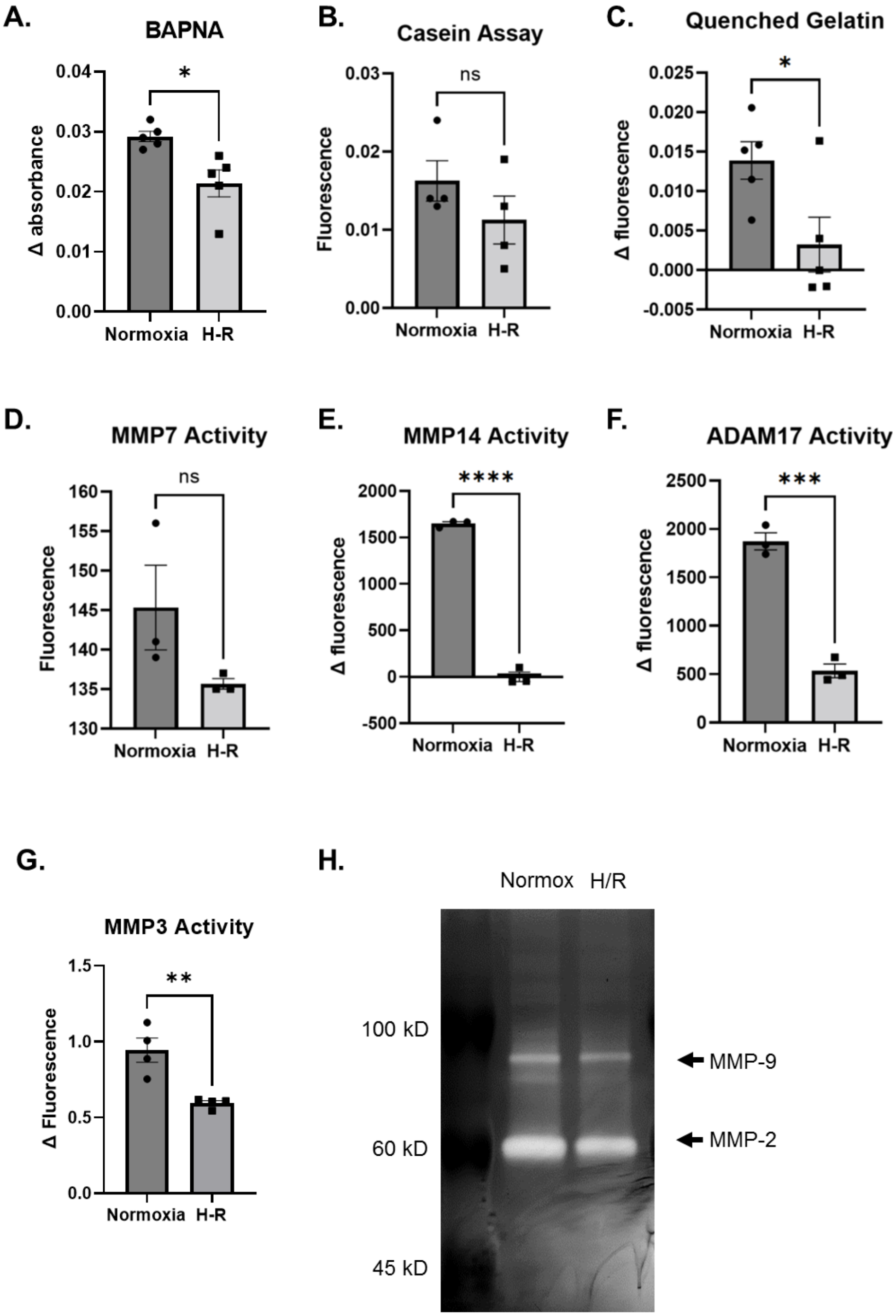
A.
BAPNA
Δ absorbance
0.04
0.03
0.02
0.01
0.00
*
Normoxia
H-R
B.
Casein Assay
Fluorescence
0.03
0.02
0.01
0.00
ns
Normoxia
H-R
C.
Quenched Gelatin
Δ fluorescence
0.025
0.020
0.015
0.010
0.005
0.000
-0.005
*
Normoxia
H-R
D.
MMP7 Activity
Fluorescence
160
155
150
145
140
135
130
ns
Normoxia
H-R
E.
MMP14 Activity
Δ fluorescence
2000
1500
1000
500
0
-500
****
Normoxia
H-R
F.
ADAM17 Activity
Δ fluorescence
2500
2000
1500
1000
500
0
***
Normoxia
H-R
G.
MMP3 Activity
Δ Fluorescence
1.5
1.0
0.5
0.0
**
Normoxia
H-R
H.
Normox
H/R
100 kD
60 kD
45 kD
MMP-9
MMP-2

Figure S6

Supplemental Fig. S6: Extracellular protease activity measurements in HUVECs.
A. Assessment of cell surface and extracellular protease activity using broad spectrum indicator BAPNA (Nα-Benzoyl-L-arginine 4-nitroanilide hydrochloride). Significance was assessed with two-tailed Student's T test. n = 5 biological replicates. **B.** Assessment of cell surface and extracellular protease activity using broad spectrum indicator succinylated casein. Significance was assessed with two-tailed Student's T test. n = 4 biological replicates. **C.** Assessment of cell surface and extracellular gelatinase activity using fluorescent quenched gelatin. Significance was assessed with two-tailed Student's T test. n = 5 biological replicates. **D.** Assessment of cell surface and extracellular MMP7 activity using fluorescent quenched substrate peptide. Significance was assessed with two-tailed Student's T test. n = 3 biological replicates. **E.** Assessment of cell surface and extracellular MMP14 activity using fluorescent quenched substrate peptide. Significance was assessed with two-tailed Student's T test. n = 3 biological replicates. **F.** Assessment of cell surface and extracellular ADAM17 activity using fluorescent quenched substrate peptide. Significance was assessed with two-tailed Student's T test. n = 3 biological replicates. **G.** Assessment of cell surface and extracellular MMP3 activity using fluorescent quenched substrate peptide. Significance was assessed with two-tailed Student's T test. n = 4 biological replicates. **H.** Gelatin zymography of media supernatant of HUVECs exposed to normoxia or H-R. MMP9 and MMP2 activity is identified using molecular weight markers. Error bars in all figures represent the mean ± s.e.m.

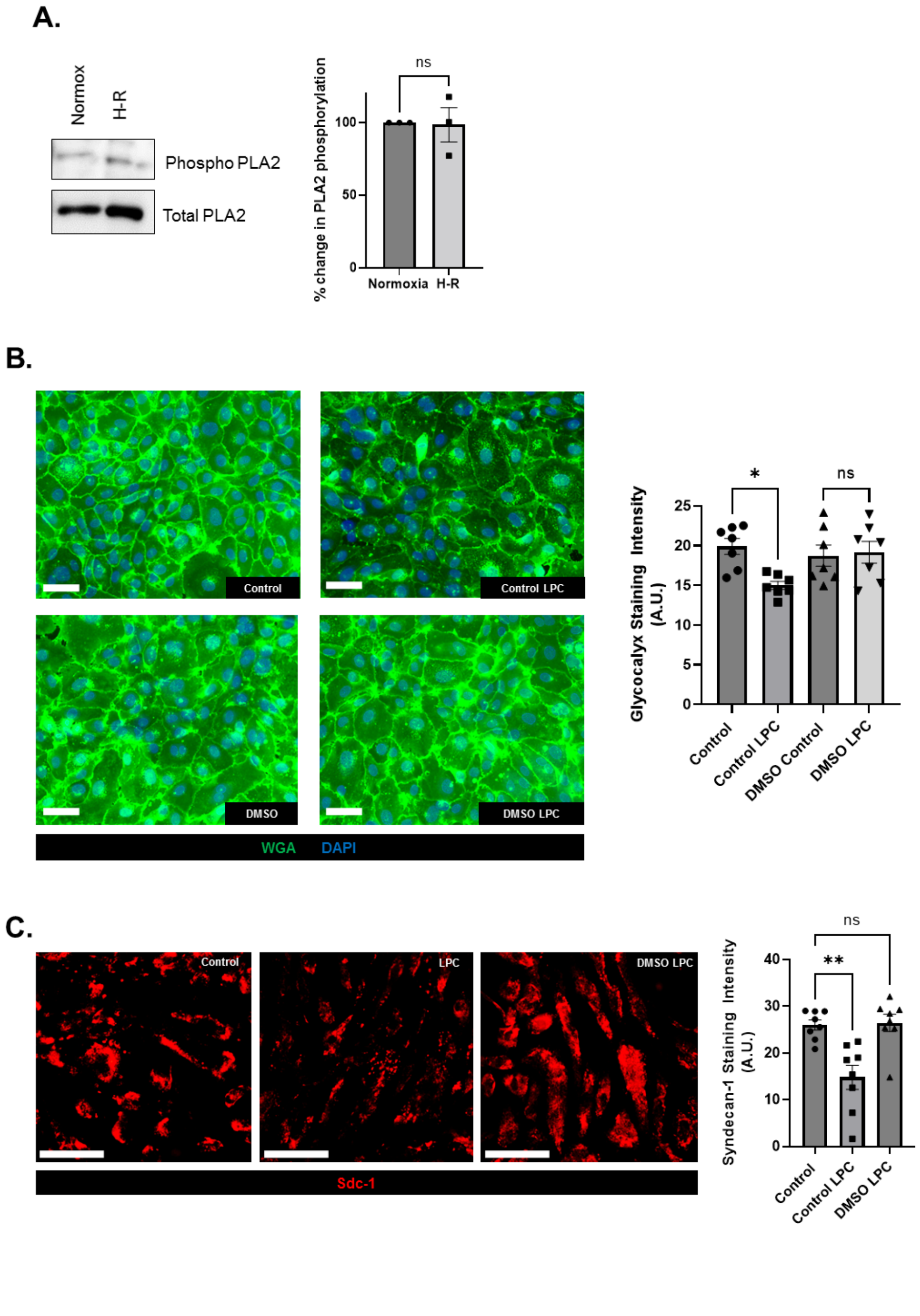
A.
Normox
H-R
Phospho PLA2
Total PLA2
% change in PLA2 phosphorylation
ns
100
50
0
Normoxia
H-R
B.
Control
Control LPC
DMSO
DMSO LPC
WGA
DAPI
Glycocalyx Staining Intensity (A.U.)
*
ns
25
20
15
10
5
0
Control
Control LPC
DMSO Control
DMSO LPC
C.
Control
LPC
DMSO LPC
Sdc-1
Syndecan-1 Staining Intensity (A.U.)
ns
**
40
30
20
10
0
Control
Control LPC
DMSO LPC

Figure S7

Supplemental Fig. S7: PLA2 phosphorylation during Hypoxia-reoxygenation and lysophosphatidylcholine-induced glycocalyx shedding.

A. Western blot of phosphorylated Phospholipase A2 and total phospholipase A2 levels from HUVECs exposed to normoxia or hypoxia-reoxygenation (H-R). Bar graph indicates densiometry quantification from blots, demonstrating no significant increase in PLA2 phosphorylation levels. Significance was assessed with two-tailed Student's T test. n = 3 biological replicates. **B.** Lysophosphatidylcholine (1 ng/ul) induces glycocalyx damage as assessed by fluorescent wheat-germ agglutinin (WGA) staining in cultured HUVECs. Dimethylsulfoxide (DMSO, 0.1 %) addition to media prevented this effect. Representative images from 7 biological replicates shown. Scale bars = 30 µm**.** **C.** Lysophosphatidylcholine (1 ng/ul) induces glycocalyx damage as assessed by fluorescent wheat-germ agglutinin (WGA) staining in cultured HUVECs. Dimethylsulfoxide (DMSO, 0.1 %) addition to media prevented this effect. Representative images from 8 biological replicates shown. Scale bars = 30 µm**.** Error bars represent the mean ± s.e.m. Significance was assessed with one-way ANOVA corrected for multiple comparisons using Tukey's method.

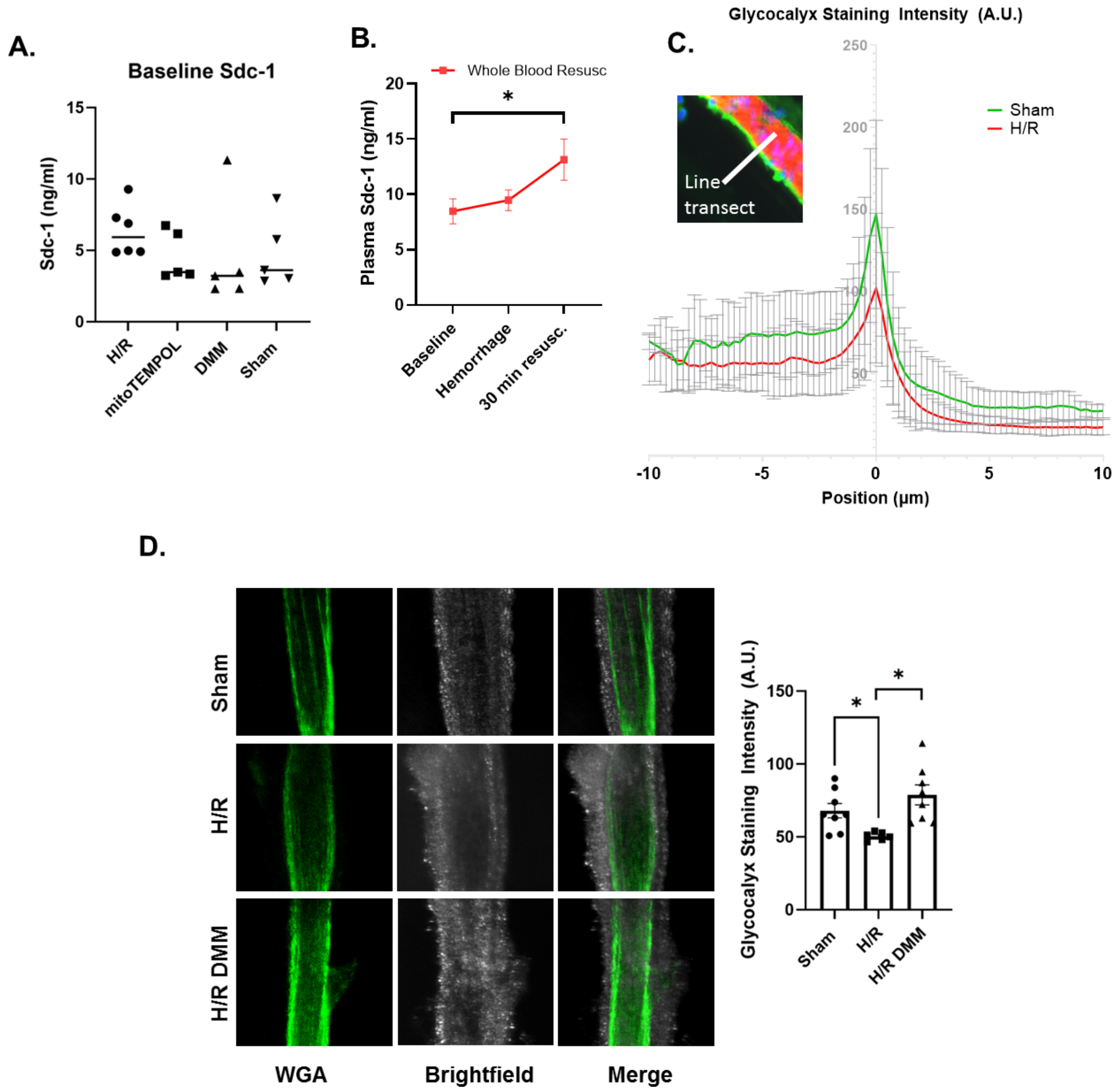
A.
Baseline Sdc-1
Sdc-1 (ng/ml)
H/R
mitoTEMPOL
DMM
Sham
B.
Whole Blood Resusc
Plasma Sdc-1 (ng/ml)
Baseline
Hemorrhage
30 min resusc.
C.
Glycocalyx Staining Intensity (A.U.)
Line
transect
Sham
H/R
Position (μm)
D.
Sham
H/R
H/R DMM
WGA
Brightfield
Merge
Glycocalyx Staining Intensity (A.U.)

Figure S8

Supplemental Fig S8: Additional data for rat model of hemorrhage.

A. Baseline plasma syndecan-1 levels from animals subjected to hemorrhage/resuscitation (H/R) or Sham protocols +/- mitoTEMPOL (5 mg/kg) or dimethylmalonate (DMM, 50 mg/kg). **B.** Plasma syndecan-1 levels from animals subjected to hemorrhage/resuscitation (H/R) protocol resuscitated using whole blood. n = 3 biological replicates. Significance was assessed with 1-way repeated measures ANOVA corrected for multiple comparisons using Tukey's method. **C.** Fluorescence levels across line transects of glycocalyx layer in Sham or H/R rats. Traces with standard deviations shown in graph. Peak glycocalyx level was set to position 0, with lumen position to the right of the origin. **D.** Imaging and quantification of pulmonary artery glycocalyx in living isolated arteries after Sham or hemorrhage/resuscitation (H/R) protocol +/- dimethylmalonate (DMM, 50 mg/kg). Fluorescent wheat germ agglutinin (WGA) used to stain glycocalyx. Significance was assessed with 1-way ANOVA corrected for multiple comparisons using Tukey's method. n = 8 arteries from 3 biological replicate animals. Error bars in all figures represent the mean ± s.e.m.

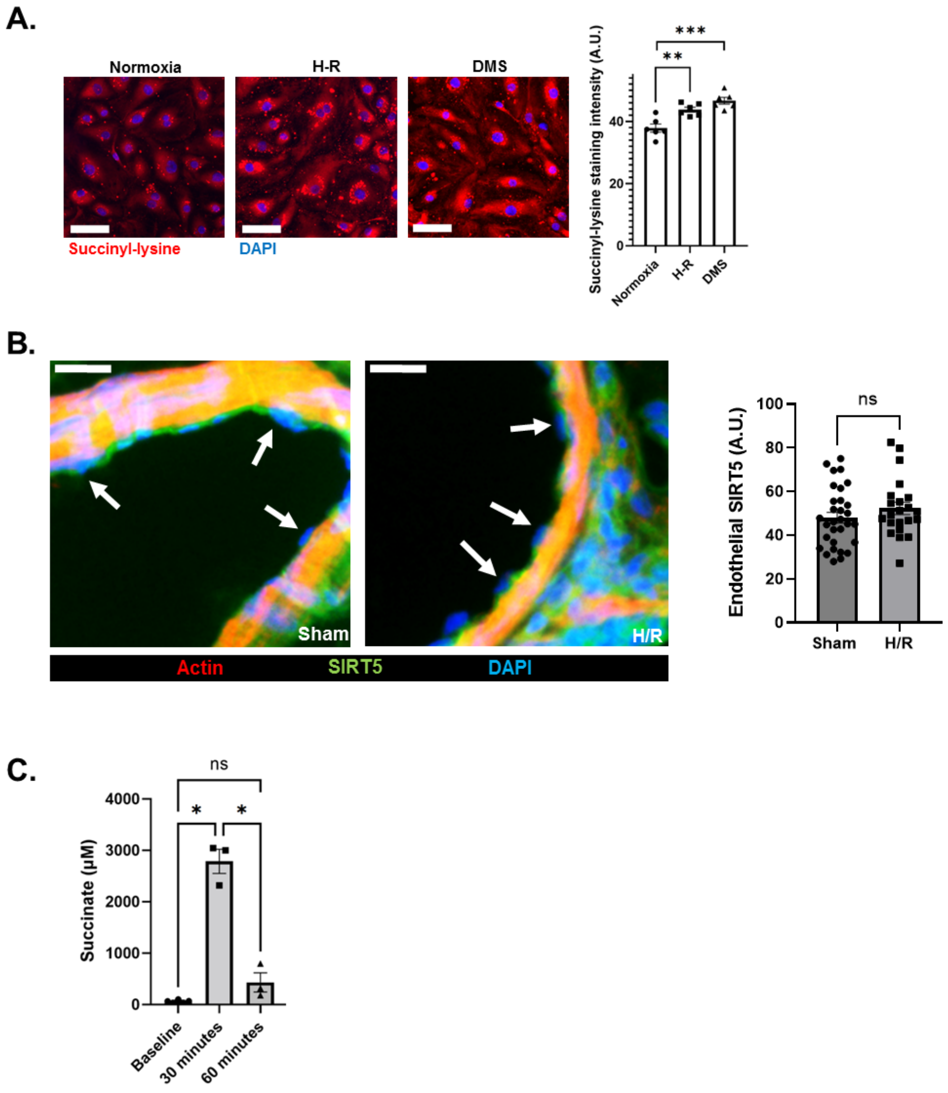
A.
Normoxia
H-R
DMS
Succinyl-lysine
DAPI
Succinyl-lysine staining intensity (A.U.)
***
**
0
20
40
Normoxia
H-R
DMS
B.
Sham
H/R
Actin
SIRT5
DAPI
Endothelial SIRT5 (A.U.)
ns
0
20
40
60
80
100
Sham
H/R
C.
Succinate (µM)
ns
*
*
0
1000
2000
3000
4000
Baseline
30 minutes
60 minutes

Figure S9

Supplemental Fig S9: Additional succinate-related data for rat model of hemorrhage.
A. Assessment of succinyl-lysine levels by immunofluorescence staining in HUVECs after hypoxia-reoxygenation (H-R) protocol or treatment with dimethylsuccinate (DMS, 50 µM). n = 6 biological replicates. Significance was assessed with 1-way ANOVA corrected for multiple comparisons using Tukey's method**.** Scale bar = 60 µM**. B.** Sirtuin 5 (SIRT5) immunofluorescence of pulmonary vessels from Sham and H/R rats demonstrate no difference in expression level. Actin stain (fluorescent phalloidin) used to aid in vessel identification. n = 22-30 cells from 4 animals per treatment group. Arrows indicate endothelial cells. Scale bar = 10 µm. Significance was assessed with two-tailed Student's T test. **C.** Plasma succinate levels measured from succinate injection rats shown in Fig. 6, assessed before injection (Baseline), and 30 and 60 minutes after injection. n=3 biological replicates. Significance was assessed with 1-way ANOVA corrected for multiple comparisons using Tukey's method.

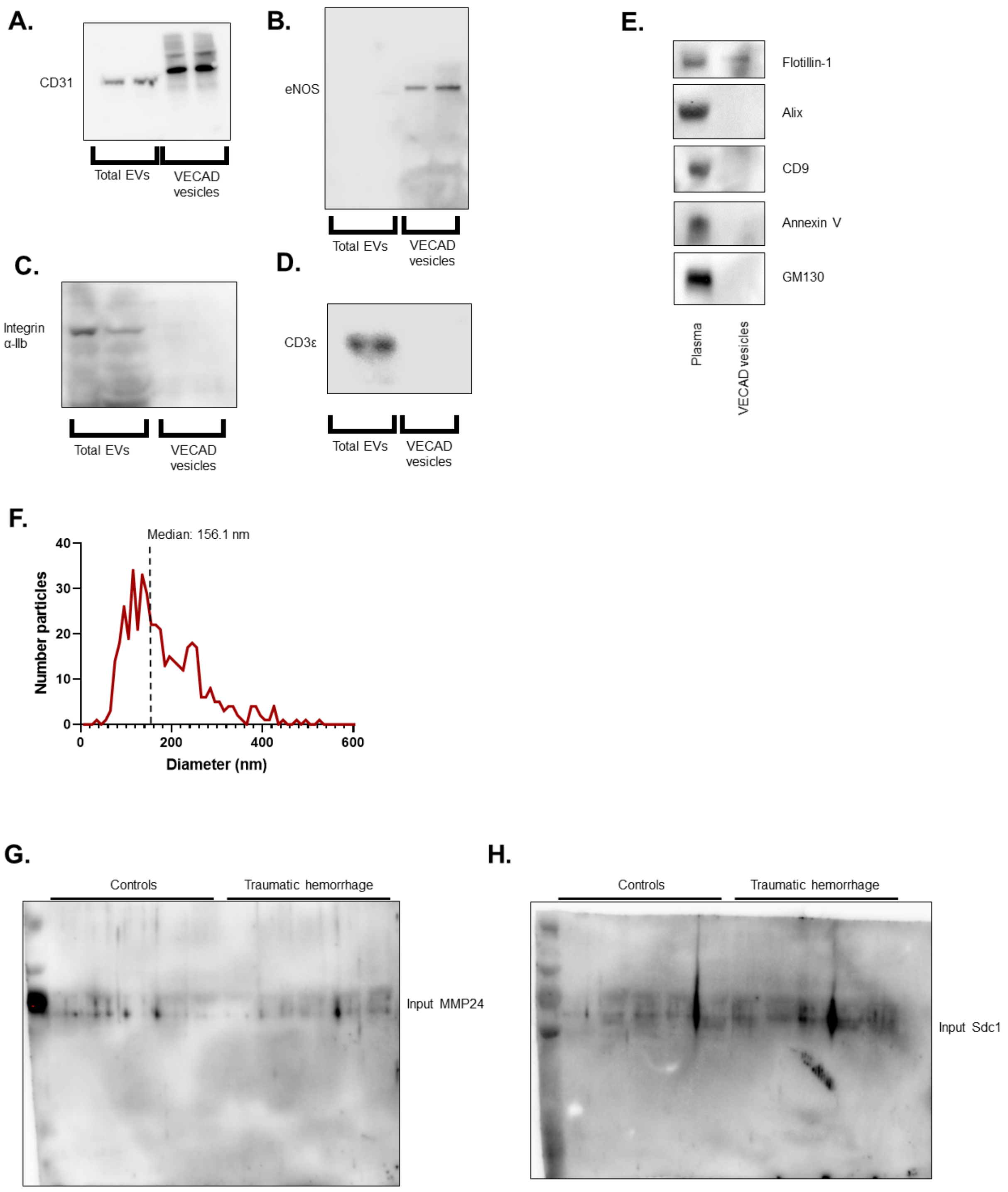
A.
CD31
Total EVs
VECAD vesicles
B.
eNOS
Total EVs
VECAD vesicles
C.
Integrin α-IIb
Total EVs
VECAD vesicles
D.
CD3ε
Total EVs
VECAD vesicles
E.
Flotillin-1
Alix
CD9
Annexin V
GM130
Plasma
VECAD vesicles
F.
Median: 156.1 nm
Number particles
Diameter (nm)
0
10
20
30
40
200
400
600
G.
Controls
Traumatic hemorrhage
Input MMP24
H.
Controls
Traumatic hemorrhage
Input Sdc1

Figure S10

Supplemental Fig S10: Trauma patient endothelial extracellular vesicle characterization. **A.** Western blot of CD31 levels from total extracellular vesicle (EVs) and purified VE-Cadherin (VECAD) vesicles from control plasma. **B.** Western blot of endothelial nitric oxide synthase (eNOS) levels from total extracellular vesicle (EVs) and purified VECAD vesicles from control plasma. **C.** Western blot of platelet marker integrin α-IIb levels from total extracellular vesicles (EVs) and purified VECAD vesicles from control plasma. **D.** Western blot of T-cell marker CD3ε levels from total extracellular vesicles (EVs) and purified VECAD vesicles from control plasma. VECAD vesicles were positive for endothelial markers CD31 and eNOS, but negative for platelet marker integrin α-IIb and T-cell marker CD3ε, supporting endothelial membrane origin. **E.** Western blot characterization of purified VECAD vesicles. Exosome markers assessed were flotillin-1, alix, and CD9. Golgi marker assessed was GM130. Microvesicle marker was Annexin V. VECAD vesicles contained lipid raft protein flotillin-1 but no classical exosome markers, and contained no organelle (golgi) proteins. **F.** Purified patient VECAD+ vesicle size quantification. **G.** Input levels of MMP24 for immunoprecipitation shown in Fig. 6H. **H.** Input levels of syndecan-1 (Sdc-1) for immunoprecipitation shown in Fig. 6H.

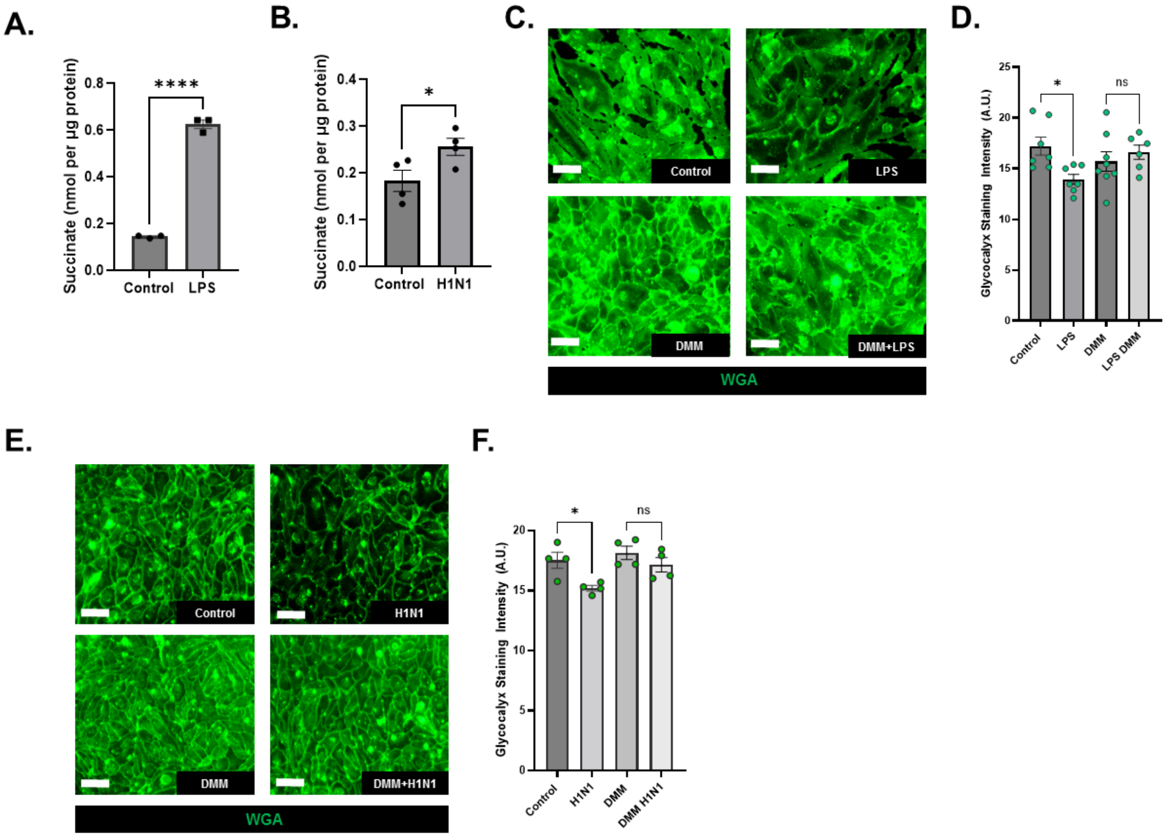

A.
Succinate (nmol per µg protein)
0.8
0.6
0.4
0.2
0.0
****
Control
LPS
B.
Succinate (nmol per µg protein)
0.4
0.3
0.2
0.1
0.0
*
Control
H1N1
C.
Control
LPS
DMM
DMM+LPS
WGA
D.
Glycocalyx Staining Intensity (A.U.)
25
20
15
10
5
0
*
ns
Control
LPS
DMM
LPS DMM
E.
Control
H1N1
DMM
DMM+H1N1
WGA
F.
Glycocalyx Staining Intensity (A.U.)
20
15
10
5
0
*
ns
Control
H1N1
DMM
DMM H1N1

Figure S11

Supplemental Figure S11: Involvement of succinate metabolism in glycocalyx damage due to lipopolysaccharide and influenza H1N1. A. Cell lysate succinate measurements from HUVECs under control or lipopolysaccharide (LPS, 1 μg/ml) exposure for 24 hours. **B.** Cell lysate succinate measurements from HUVECs under control or H1N1 (multiplicity of infection = 5) exposure for 24 hours. **C.** Surface FITC-wheat germ agglutinin (WGA) staining during control or 24 hour LPS exposure to assess glycocalyx levels in HUVECs +/- dimethylmalonate (DMM, 50 μM). **D.** Quantification of staining intensity from experiments in C. **E.** Surface FITC-wheat germ agglutinin (WGA) staining during control or 24 hour H1N1 exposure to assess glycocalyx levels in HUVECs +/- dimethylmalonate (DMM, 50 μM). **F.** Quantification of staining intensity from experiments in E. Significance was assessed with two-tailed Student's T test (A, B) or 1-way ANOVA corrected for multiple comparisons using Tukey's method (D, F). Error bars in all figures represent the mean ± s.e.m.

Table S1. Demographics of Trauma Patient Study Population (n=50)

Characteristics	Mean (or n)	Standard Deviation
Male sex n	41 (82%)	-
Age (years)	41.1	19.3
Penetrating injury n	21 (42%)	-
Prehospital GCS	11.7	4.6
Prehospital fluids (L)	0.53	0.86
Time since injury (min)	49.1	27.1
Shock index	1.08	0.64
Prothrombin time (PT, sec)	13.5	3.9
Plasma succinate (μM)	245.1	208.3
Plasma syndecan-1 (ng/ml)	60.5	85.9
Fibrinogen (mg/dL)	370.3	99.1
Lactate (mmol/L)	3.76	2.91
Potassium (mmol/L)	3.8	0.7
$HCO3^-$ (mEq/L)	21.8	3.8

GCS=Glasgow Coma Scale. Shock index = (systolic blood pressure)/(heart rate).
